# Phase-specific and laser-modulated polydopamine-chlorella-curdlan hydrogels: pioneering a melanoma integrative therapy

**DOI:** 10.7150/thno.113417

**Published:** 2025-06-23

**Authors:** Zhenzhen Yan, Tinglin Zhang, Yuxiang Wang, Chao Ji, Heru Wang, Yicheng Ma, Jingzhu Li, Yixin Wu, Zhi Liu, Jingnan Xun, Xiaowan Fang, Yongjun Zheng, Shizhao Ji, Shichu Xiao, Jie Gao

**Affiliations:** 1 Department of Burn Surgery, The First Affiliated Hospital of Naval Medical University, Shanghai 200433, P. R. China.; 2 Changhai Clinical Research Unit, Shanghai Changhai Hospital, Naval Medical University, Shanghai 200433, P. R. China.; 3 Neurovascular Center, The First Affiliated Hospital of Naval Medical University, Shanghai 200433, P. R. China.; 4 Department of Dermatology, The First Affiliated Hospital of Naval Medical University, Shanghai 200433, P. R. China.; 5 Shanghai Key Laboratory of Nautical Medicine and Translation of Drugs and Medical Devices, Shanghai 200433, P. R. China.; Zhenzhen Yan, Tinglin Zhang, Yuxiang Wang and Chao Ji contributed equally to this paper.

**Keywords:** melanoma, wound healing, hypertrophic scar, hydrogels, integrative therapy

## Abstract

**Background:** Patients with melanoma are confronted with postoperative challenges such as tumor relapse, impaired wound healing, infection, and hypertrophic scarring, with chronic wound inflammation potentially increasing the risk of tumor recurrence. Photoresponsive hydrogels have great potential for integrated postsurgical treatment of melanoma by embedding photosensitizers or photothermal agents in porous polymer networks, combining the advantages of hydrogels and phototherapy technologies.

**Methods:** The photoresponsive PCCUR hydrogel was synthesized via a thermal induction method, incorporating polydopamine nanoparticles (PDA-NPs), *Chlorella* extract (CE), and curdlan (CUR). The physicochemical properties of PCCUR and its effects on skin cells and B16F10 cells were systematically evaluated through in vitro experiments. The antitumor recurrence efficacy of the PCCUR hydrogel was assessed via an incomplete melanoma resection model. Furthermore, the regulatory effects of PCCUR on infected wound healing and its inhibitory effects on hypertrophic scar formation were investigated under temporally controlled laser irradiation.

**Results:** The photoresponsive PCCUR hydrogel possesses temperature sensitivity, injectable properties, optimal mechanical strength, and significant antimicrobial activity and is tailored to mitigate postsurgical issues through healing phase-specific laser irradiation adjustments. During the inflammatory phase, synergistic photodynamic therapy (PDT) and photothermal therapy (PTT) not only eliminate infections but also induce immunogenic cell death (ICD), releasing tumor antigens that bypass tumor heterogeneity and are captured by the *in situ* hydrogel. The antigen-captured hydrogel acts as an “antigen reservoir” and releases captured antigens to recruit dendritic cells (DCs) to stimulate an antitumor immune response. In the proliferation phase, the inherent properties of the hydrogel inhibit inflammation and facilitate cell proliferation and angiogenesis. The remodeling phase involves low-intensity laser-based phototherapy to modulate myofibroblast activity and collagen remodeling, preventing hypertrophic scarring.

**Conclusions:** The dynamic photoresponsive properties of PCCUR by introducing a 3R paradigm (Remove-Residual tumor cells/bacteria; Remodel-Immune microenvironment; Repair-Skin tissue) provide a safe and efficient therapeutic approach tailored to the three stages of wound healing, making it a promising dressing option for the comprehensive management of postoperative wounds in melanoma patients.

## Introduction

**Figure 1:**
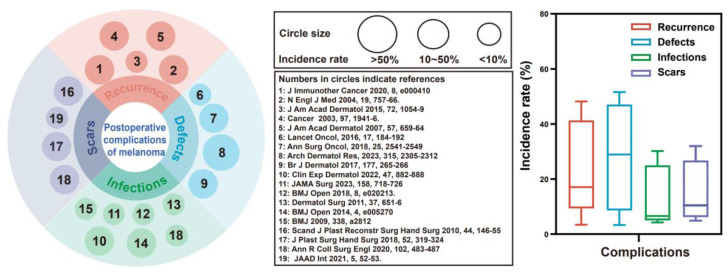
** Clinical data on complications after resection of skin cancer.** The left panel displays circles of varying sizes representing reported incidence rates of complications: small circles indicate an incidence rate of <10%, medium circles denote rates between 10% and 50%, and large circles correspond to rates of >50%. The number within each circle refers to the relevant reference cited in the center. The right panel presents a box plot illustrating the distribution range and median incidence rates for each of the four major complications associated with skin cancer resection.

Cutaneous melanoma, one of the most aggressive and fatal forms of skin cancer, is a malignant tumor arising from melanocytes in the skin and has a steadily increasing global incidence 1. In 2020, an estimated 325,000 new cases were identified globally. If current trends continue, this figure is expected to increase by more than 50% by 2040, posing a substantial and ongoing public health challenge 2. Although early-stage melanomas benefit from wide local excision, nearly 50% of high-risk stage II patients still experience recurrence or metastasis 3, 4. We reviewed 19 studies involving 6,301 patients with skin cancer, which identified four major challenges following surgical excision of skin cancer: 1. Tumor recurrence (range: 3.4-48.1%, median: 17.1%): Incomplete surgical resection inevitably increases the risk of recurrence or metastasis 5-7. 2. Cutaneous defects (range: 3.3-51.6%, median: 28.9%): Extensive local excision often results in substantial skin defects, necessitating repair and reconstruction 8, 9. 3. Wound infection (range: 4.2-30.2%, median: 6.6%): Postoperative infection risks are increased due to large skin defects, bacterial colonization, and immunocompromised patients 10, 11. 4. Scar hypertrophy (range: 4.9-32.0%, median: 10.5%): A chronic inflammatory microenvironment and overactivation of myofibroblasts increase susceptibility to scar formation during the process of wound healing 12 (**Figure 1, Supplementary Table 1**). Therefore, surgical excision is the primary treatment for melanoma, but it faces challenges such as incomplete tumor removal and potential skin defects. These issues can lead to tumor recurrence, metastasis, infections, and chronic wounds. Notably, inflammation in chronic wounds may promote mutant cell growth and increase the risk of hypertrophic scarring and tumor recurrence 13. Therefore, there is an urgent need for a multifunctional therapeutic material capable of mitigating these complications.

The Chinese Anti-Cancer Association (CACA) has emphasized the importance of protecting skin and mucosal integrity during cancer treatment, as it significantly enhances patient compliance and overall quality of life 14. In alignment with this, our research advocates for an integrated therapeutic strategy that maximizes the effectiveness of comprehensive cancer treatment while facilitating personalized therapeutic regimens 15-17. Compared with the combined use of separate antitumoral and reparative materials, integrated materials offer substantial benefits, including improved synergy, convenience, and safety in addressing melanoma-related postoperative complications 18, 19: 1. Synergy: Anti-tumoral interventions can minimize tissue damage and promote tissue repair, while tissue regeneration could also improve patient tolerance to tumor therapies. 2. Convenience: Integrated materials streamline treatment procedures, reducing patient burden. 3. Safety: By minimizing the introduction of exogenous materials, integrated materials lower the risk of adverse toxic effects. Phototherapy materials, such as photothermal and photodynamic agents, can increase the temperature or generate reactive oxygen species (ROS) under specific light sources, thus achieving photothermal therapy (PTT) and photodynamic therapy (PDT) 20-22. Our previous studies developed two innovative integrated phototherapy agents: a tumor cell membrane-decorated Prussian blue nanovaccine (PBVac) and polydopamine-modified porous poly(lactic‒coglycolic acid) microspheres encapsulating a copper-based metal-organic framework coated with a tumor cell membrane (PLGA/PDA-CCM) that activate antitumor immunity while promoting tissue repair 23, 24. Despite their therapeutic potential, these materials face limitations related to biosafety, complexity, and rapid clearance.

While individual applications of PDT or PTT have been shown to be suboptimal in achieving robust antitumor responses, a combined therapeutic approach harnessing both modalities has demonstrated synergistic potential. PTT increases oxygen levels and photosensitizer uptake, increasing the effectiveness of PDT. PDT, in turn, can target heat-resistant tumors, enhancing the potential of PTT 25-27. In addition, the combined PDT/PTT strategy also shows great potential as an antimicrobial 28. However, the continued generation of heat and ROS causes nonspecific loss of normal tissue 29, 30. The growing interest in using low-level laser therapy (LLLT) for tissue repair is noteworthy 31-33. On the basis of prior research, we propose that LLLT may facilitate scar-free healing after tumor resection.

*Chlorella*, a green microalga, has garnered significant attention for its therapeutic potential as a photosensitizer in PDT. The intrinsic chlorophyll within *Chlorella* not only participates in photosynthesis to generate oxygen but also serves as a natural photosensitizer 34-37. *Chlorella* extract (CE), which is rich in chlorophyll, proteins, and polysaccharides, has demonstrated photosensitizer activity and antioxidant and anti-inflammatory properties 38, 39. Compared with that of live *Chlorella*, the ease of production of CE, which is devoid of complex cultivation, renders it cost-effective for large-scale applications 40. Consequently, CE has potential as a safe and effective PDT agent for both tumor inhibition and tissue repair. Polydopamine nanoparticles (PDA-NPs), synthesized from the natural neurotransmitter dopamine, have attracted attention for their multifunctionality in tumor therapy and wound healing. Their key advantages include excellent biocompatibility, high photothermal conversion efficiency, strong adhesiveness for tumor antigen adsorption, and intrinsic anti-inflammatory and antioxidant properties, all of which contribute to enhanced therapeutic outcomes 23, 41-44.

**Scheme 1:**
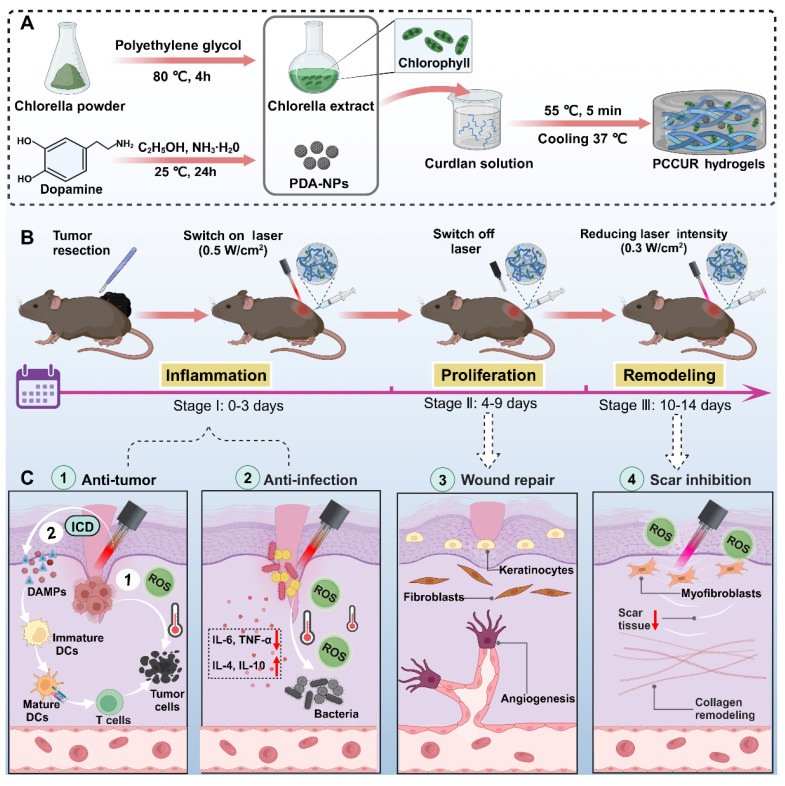
** Schematic representation of the application of PCCUR in managing postoperative complications in patients with melanoma.** (A) Synthesis of PCCUR *via* physical blending, emphasizing its constituent components and the fabrication process. (B) Integrated therapeutic approach utilizing PCCUR to manage postoperative complications by modulating the laser intensity at various stages of wound healing following melanoma resection. (C) PCCUR employs a multifaceted mechanism to provide an integrated treatment approach, encompassing antitumor activity, anti-infection properties, the enhancement of wound repair, and the inhibition of scar formation. CE (*Chlorella* extract), PDA-NPs (polydopamine nanoparticles), CUR (curdlan), DCs (dendritic cells), ROS (reactive oxygen species), ICD (immunogenic cell death), DAMPs (damage-associated molecular patterns), TNF-α (tumor necrosis factor-alpha), and IL-6 (interleukin-6).

Extending the retention time of therapeutics in tumor and wound sites is crucial for strengthening antitumor actions, reducing recurrence, and facilitating wound repair 45. Our team identified hydrogels—polymeric, hydrophilic, 3D networks—as effective vehicles for the sustained release of nanomaterials, promoting enhanced healing 46, 47. In particular, injectable hydrogels are valued for their biocompatibility, biodegradability, and tunable physical properties, which enable precise drug targeting to bacterial and cancerous tissues 48. Curdlan (CUR), an unbranched β-(1,3)-D-glucan, is favored in both the food industry and biomedical fields because of its biosafety and degradability. Upon heating to 55-80 °C, CUR forms a thermoreversible gel, making it an ideal candidate for *in situ* gelation and controlled drug release 49. However, its applications in cutaneous wound healing remain largely unexplored. To address the challenges of melanoma surgical complications, our study introduces a hydrogel system, PCCUR (PDA-NP- and CE-incorporated CUR hydrogels), engineered to respond to dynamically modulated laser intensities, providing a customized therapeutic strategy throughout the wound healing process (**Scheme 1**). During the inflammatory phase, high-intensity laser irradiation is utilized to synergistically activate PDT and PTT, eliminating residual tumor cells and pathogens. This process embodies the “Remove” principle. Furthermore, PCCUR adsorbs antigens released during PDT/PTT-induced immunogenic cell death (ICD) of tumor cells. This action demonstrates the “remodeling” principle by reshaping the immune microenvironment to enhance antitumor immunity. During the proliferation phase, the properties of PCCUR promote cell proliferation and angiogenesis, which are essential for effective wound healing. The remodeling phase is carefully managed with low-intensity laser therapy to regulate collagen remodeling and prevent hypertrophic scarring. This final phase exemplifies the “Repair” principle by promoting tissue regeneration and minimizing scar formation. The PCCUR's multifunctional design presents a comprehensive treatment strategy for melanoma, uniquely addressing antirecurrence, infection control, wound repair, and scar prevention. The 3R paradigm (Remove residual tumors/bacteria; Remodel immune microenvironment; Repair skin tissue) addresses critical challenges through a unified platform that simultaneously prevents recurrence, accelerates recovery, and minimizes scarring. The multifunctional design of the PCCUR presents a comprehensive treatment strategy for melanoma through a single laser source, marking a significant advancement in melanoma therapy. The excellent biocompatibility and biosafety of PCCUR indicate its potential for clinical application, as it is promising for enhancing patient outcomes and quality of life by revolutionizing postoperative melanoma care.

## Methods

### Cell culture

The B16F10 and human umbilical vein endothelial cells (HUVECs) cell lines were cultured in high-glucose Dulbecco's modified Eagle's medium (DMEM) enriched with 10% FBS and 1% antibiotics (penicillin/streptomycin). Hypertrophic scar fibroblasts (HSFs) and human fibroblasts (HFBs) are derived from the enzymatic digestion of surgically excised hypertrophic scar tissue and adjacent healthy skin via dispase II and collagenase. The isolated HSFs were maintained in DMEM containing 10 ng/mL tumor growth factor-beta 1, while the HFBs were cultured in DMEM supplemented with 10% FBS and 1% penicillin‒streptomycin. Cells between passages 2 and 5 were selected for subsequent experiments. All the cells were maintained at 37 °C in a humidified atmosphere with 5% CO₂.

### Preparation of CE

*Chlorella* powder (0.2 g; G-03, Zhenghe Pharmaceutical Co., Ltd., Shanxi, China) was deposited into a cleaned and dry round-bottomed flask. Next, 20 mL of polyethylene glycol (PEG) 200 (P103718, Aladdin, Shanghai, China) was added to the flask. The mixture was vigorously stirred magnetically in an 80 °C water bath for 4 h in the dark. Following extraction, the samples were centrifuged at 10,000 rpm for 10 min and filtered through a 0.45 μm membrane to obtain CE. Chlorophyll-a is commonly used in photodynamic therapy because of its high extinction coefficient at 660 nm 50. The composition of the CE was analyzed *via* ultraviolet‒visible (UV‒Vis) absorption spectroscopy, and its chlorophyll-a content was quantified according to the chlorophyll-a standard (C805046, Macklin, Shanghai, China).

### Preparation of hydrogels

PDA-NPs were synthesized following a previously established protocol. Comprehensive procedures are available in the Supplementary Material. PDA-NPs (2 mg/mL) were ultrasonically dispersed in 900 μL of phosphate-buffered saline (PBS). Subsequently, 100 μL of CE and 45 mg of curdlan powder (CLC-C, Dongsheng Biotech Co., Ltd., Shanghai, China) were added to the above mixture, which was stirred for 3 h in an oxygen-free, dark environment. The mixture was heated to 55 °C for 5 min and then cooled to 37 °C to induce gelation, forming the PCCUR hydrogel. The CCUR hydrogel was prepared in the same manner but without the PDA-NPs. Similarly, the CUR hydrogel, which was used as a control, was prepared by replacing the CE solution with PBS.

### Characterization of the hydrogels

Lyophilized hydrogel samples were sputter-coated with gold and examined for microstructure using a Zeiss Sigma 300 scanning electron microscope (SEM, Carl Zeiss, Germany). Pore sizes were quantified *via* ImageJ software (NIH, USA). The rheological properties of the hydrogels were assessed via a rheometer (Kinexus Prime Ultra+. NETZSCH, Selb, Germany) for the time sweep and temperature sweep tests. Compression tests were conducted on the hydrogel samples (diameter, 10 mm; thickness, 5 mm) via an electronic universal testing machine (HID-B609B-S; Shanghai Hefu Medical Equipment Co. Ltd., Shanghai, China). The swelling and degradation of the CUR, CCUR, and PCCUR hydrogels were examined in PBS. The detailed procedures are provided in the Supplementary Material. For release studies, PCCUR hydrogels were incubated in 2 mL of PBS at 37 °C. At specified intervals (1, 3, 5, 7, 10, and 14 days), 10 µL aliquots were collected and replaced with an equal volume of fresh PBS. The cumulative release of the CE and PDA-NPs was monitored via UV‒Vis spectrophotometry, and the absorbances at 660 nm (CE) and 280 nm (PDA-NPs) were measured.

### Photothermal behavior

The photothermal performance of the PCCUR hydrogel was assessed via an infrared thermal imaging system (225S-L24, Fotric, Shanghai, China). Briefly, 200 μL of hydrogels with different concentrations of PDA-NPs (0, 0.5, 1, and 1.5 mg/mL) were irradiated with a 660 nm laser (FC-660-5000-MM, Shanghai Xilong Optoelectronics Technology Co., Ltd., Shanghai, China) at 0.5 W/cm² for 5 min. Thermal images and real-time temperature variations were recorded throughout the process. Additionally, the temperature profile of the PCCUR hydrogel was monitored under various laser power densities (0.3, 0.5, and 0.7 W/cm²) for 5 min by 660 nm laser irradiation. Finally, the stability of the PCCUR hydrogel under photothermal conditions was assessed by turning the laser on and off for three cycles. The efficiency of photothermal conversion of the hydrogel was determined according to the methodology in the referenced literature 51.

### Photodynamic behavior

To assess the photodynamic properties of the PCCUR hydrogel, singlet oxygen sensor green (SOSG, MA0326, MeilunBio, China) was employed for the detection of singlet oxygen (¹O₂). Briefly, 0.5 mL of SOSG solution (5 μM) was incubated with the PCCUR hydrogel. The mixture was subsequently exposed to a 660 nm laser at varying power densities for 5 min. Measurements of fluorescence intensity at 525 nm were conducted at specific time points *via* a fluorescence spectrophotometer (RF-5301, Shimadzu, Japan). To further evaluate ROS production, we utilized three distinct fluorescent probes: SOSG and DPBF (1,3-diphenylisobenzofuran) for singlet oxygen (¹O₂) detection and DHE (dihydroethidium) for superoxide anion (O₂⁻˙) detection. UV‒visible spectrophotometric analysis was performed to quantify ROS generation in different hydrogels under 660 nm (0.3 W/cm²) laser irradiation for 5 min.

### Hemolysis assay

Blood samples freshly obtained from healthy C57BL/6 mice were prepared by washing and dilution for subsequent hemolysis studies as previously described 52. The detailed procedures are provided in the Supplementary Material.

### *In vitro* cytocompatibility

Different volume fractions of CE (0-20%) were cocultured with HFBs to determine the optimal loading concentration of CE. Hydrogel extracts were prepared by incubating 100 μL of each hydrogel in 1 mL of PBS at 37 °C with shaking for 48 h. Subsequently, cells at a density of 5000/well in a 96-well plate were cultured overnight. The viability of HFBs and HUVECs was evaluated *via* a cell counting kit-8 (CCK-8, Dojindo, Kumamoto, Japan), with the absorbance measured at 450 nm with a multifunctional microplate (ULX800, BioTEK, Vermont, USA).

### Cell migration and tube formation assays

Scratch and Transwell assays were used to assess cell migration. Upon reaching 90% confluence in 6-well plates, HFB cells were uniformly scratched with a 200 μL pipette tip to mimic incisions. The cells were washed with PBS and then cultured with different hydrogel extracts or with PBS (control). Microscopy images were captured at intervals of 0, 12, and 24 h, and wound closure was measured *via* ImageJ software.

For the Transwell assay, standard Transwell chambers with an 8 μm pore size (Corning, USA) were used in 24-well plates. The upper chamber was filled with a 200 μL serum-free suspension of HUVECs (5 × 10^4^ cells/mL). The lower chamber contained 700 μL of hydrogel extracts or PBS containing 1% serum. Cells were fixed with 4% paraformaldehyde (PFA), stained with 1% crystal violet, and photographed after 24 h to evaluate migration.

For the tube formation experiments, HUVECs were incubated for 24 h with various hydrogel extracts or with PBS as a control. The cells (4 × 10^4^ cells/well) were then seeded into 96-well plates precoated with Matrigel (Corning, NY, USA). Following a 6 h incubation at 37 °C, images were taken via an inverted microscope and analyzed with ImageJ software to assess tube formation.

### *In vitro* antitumor experiments

B16F10 melanoma cells (1 × 10⁵; Shanghai Kwai Sai Biotechnology Co. Ltd., China) were seeded into 24-well plates and incubated overnight. After a 6 h incubation with different hydrogels or PBS (control), the cells were irradiated with a 660 nm laser (0.5 W/cm^2^, 5 min). Following an additional 18 h of incubation, the cell viability was determined *via* a live/dead kit (Invitrogen, USA), and fluorescence microscopy (Olympus) was employed to capture images.

### Intracellular ROS generation assays

B16F10 cells (1 × 10⁵) were seeded in 24-well plates and incubated overnight. The cells were then treated with different hydrogels or PBS (control) for 24 h. Subsequently, fresh medium containing 10 μM 2',7'-DCFH-DA was added, followed by a 30 min incubation. After being washed with PBS, the cells were exposed to a laser (660 nm, 0.5 W/cm^2^, 5 min) to stimulate ROS production. Fluorescence was detected *via* a fluorescence microscope (Olympus).

### *In vitro* induction of ICD

The evaluation of ICD was facilitated by employing a calreticulin (CRT) immunofluorescence assay, high mobility group box 1 (HMGB1) enzyme-linked immunosorbent assay (ELISA) kit, and adenosine triphosphate (ATP) assay kit, as previously described 23. The specific methodologies are described in the Supplementary Material. Briefly, B16F10 cells were seeded at 5 × 10⁴ cells per well in 12-well plates and cultured for 12 h. Subsequently, different hydrogels or PBS (as a control) were administered for 24 h. For the laser irradiation groups, cells were exposed to a 660 nm laser (0.5 W/cm², 5 min), followed by 24 h of incubation for assessment.

### *In vitro* apoptosis assays

After HFBs were induced to become fibrotic by DMEM containing 10 ng/mL transforming growth factor beta 1 (TGF-β1), they were inoculated into 6-well plates and treated with the PCCUR hydrogel or PBS (control group) for 24 h. The laser group was irradiated with a 660 nm laser (0.3 W/cm^2^) for 5 min. Treated cells were stained with an Annexin V-APC/7-AAD apoptosis kit (MultiSciences, Hangzhou, China) and incubated for 5 min following the manufacturer's guidelines. The fluorescence signals were monitored and analyzed *via* a Beckman Coulter flow cytometer (CA, USA).

### *In vitro* antibacterial assays

To assess the antibacterial properties of the hydrogels, 100 µL of each hydrogel or PBS (as a control) was introduced into 1 mL of *S. aureus* or* E. coli* solutions (1 × 10⁶ CFU/mL) in centrifuge tubes. The mixtures were either exposed to a 660 nm (0.5 W/cm²) laser for 5 min or stored in the dark. The solution was then diluted 100-fold, and 20 µL of the diluted bacterial suspension was spread onto agar plates. These plates were incubated for 18 h at 37 °C to observe the colonies. Bacterial viability was evaluated using bacterial live/dead kit (Invitrogen, USA), followed by imaging *via* fluorescence microscopy. SEM was employed to examine bacterial morphology.

For biofilm formation, a methicillin-resistant *S. aureus* (MRSA, 1 × 10^7^ CFU/mL) suspension was incubated in a 24-well plate at 37 °C, and the medium was changed every 24 h for a total incubation of 48 h to facilitate biofilm formation. After the supernatant was removed, the biofilms on the well bottoms were treated with different hydrogels followed by laser irradiation. The biofilms were then fixed with anhydrous methanol for 30 min, stained with 0.1% crystal violet for 30 min, and then gently washed with PBS. Images were captured, and the biofilms were subsequently washed with 30% acetic acid to solubilize the dye. The OD value at 495 nm was measured to quantify the biofilm biomass.

### *In vivo* biocompatibility of the hydrogels

All animal experiments were approved by the Animal Research Ethics Committee at Shanghai Changhai Hospital (Shanghai, China). The approval number was 2024-021. The C57BL/6 mice used in this study were all purchased from SPF (Beijing) Biotechnology Co., Ltd. (Beijing, China). To evaluate the *in vivo* degradation and biocompatibility of the hydrogel, sterilized CUR, CCUR, and PCCUR hydrogels were implanted subcutaneously into C57BL/6 mice (female, 6-8 weeks old). After isoflurane-induced anesthesia, surgical incisions were created on the dorsal skin, allowing for hydrogel implantation. Skin tissue samples were harvested at postoperative days 3, 7, 10, and 14 for hematoxylin‒eosin (H&E) staining to assess hydrogel degradation. At the 14-day endpoint, blood was collected *via* enucleation for routine blood, liver function, and kidney function tests. Major organs, including the heart, liver, spleen, lungs, and kidneys, were collected and subjected to H&E staining.

### *In vivo* prevention of tumor recurrence

A subcutaneous B16F10 melanoma model was developed by injecting 5 × 10^5^ B16F10 cells in 100 μL of PBS into the right flank of C57BL/6 mice (female, 5-6 weeks). When the tumor volume reached approximately 150-200 mm^3^, 90% of the tumor tissue was resected, and 5% of the tumor tissue was retained to simulate an incompletely resected tumor recurrence model. The mice were randomly assigned to six groups (n = 5): control, control + laser, CCUR, CCUR + laser, PCCUR, and PCCUR + laser. All the laser treatment groups were immediately subjected to laser exposure (660 nm, 0.5 W/cm², 5 min) following hydrogel injection. During the 14-day treatment period, laser treatment was administered every two days. The tumor size and mouse weight were recorded every two days. The tumor volume was calculated via the following formula: Volume = (a × b^2^)/2, where 'a' is the longest diameter and 'b' is the shortest diameter. A humane endpoint of tumor volume exceeding 2000 mm³ was established for the animal experiments. On day 14, the mice were euthanized, and their tumors and organs were collected, fixed in 4% PFA, and subjected to histological analysis *via* H&E staining, immunofluorescence, and immunohistochemistry.

To elucidate the immunotherapeutic mechanisms of PCCUR, we performed flow cytometric analysis on TDLNs and spleens from mice on day 14 after treatment. Single-cell suspensions were obtained by mechanically homogenizing TDLN and spleen tissues. To identify mature DCs, the cells were stained with the following antibodies: PerCP/Cyanine 5.5-conjugated anti-CD45, FITC-conjugated anti-CD11c, APC-conjugated anti-CD80, and PE-conjugated anti-CD86 (BioLegend, California, USA). Flow cytometry was used to analyze the stained cells and quantify the DC populations. Tumor tissues were similarly processed and stained with antibodies targeting PerCP/Cyanine5.5-conjugated anti-CD45, APC-conjugated anti-CD3, FITC-conjugated anti-CD4, and PE-conjugated anti-CD8 (BioLegend, California, USA) to assess T-cell activation.

### *In vivo* assessment of infected wounds

An infection model was developed by creating an 8 mm circular wound on the backs of C57BL/6J mice (female, 6-8 weeks), followed by inoculation of the wound with *S. aureus* (1 × 10⁷ CFU/mL, 50 μL). The mice were then randomly divided into four groups (n = 5): control, PCCUR, PCCUR + L-1, and PCCUR + L-1 + L-2. Here, L-1 represents high-intensity laser therapy (660 nm, 0.5 W/cm²) applied from day 0 to day 3, whereas L-2 refers to low-intensity laser irradiation (660 nm, 0.3 W/cm²) from day 10 to day 14. Throughout the treatment period, the wound area was photographed and measured via ImageJ software on days 0, 3, 7, and 10. The wound healing rate was calculated via the following formula: Remaining wound area (%) =S/S_0_ × 100%, where S_0_ represents the initial wound area on day 0 and S denotes the wound area at subsequent time points. Additionally, skin tissue was collected on day 3 for the detection of inflammatory factors via ELISA kits (AiFang Biological Co., Ltd. Hunan, China). Regenerative wound tissues were harvested for histological analysis through H&E staining, Masson's trichrome staining and α-smooth muscle actin (α-SMA) immunofluorescence staining.

Wound tissue was harvested on day 7 posttreatment for RNA sequencing (Biotechnology Co., Shanghai, China). Total RNA extracted from the tissues was prepared for library preparation by Biotechnology Corporation (Shanghai, China). Differentially expressed genes (DEGs) were identified *via* DESeq2 software with criteria set at a |log_2_-fold change| ≥ 1 and a Q value ≤ 0.05. Additionally, bioinformatics analysis involved performing Gene Ontology (GO) and Kyoto Encyclopedia of Genes and Genomes (KEGG) pathway enrichment analyses.

### *In vivo* evaluation of hypertrophic scars in rabbit ears

A hypertrophic scar model was developed using adult New Zealand white rabbits (2.0-2.3 kg) sourced from Jiaxing Shengwang Ecological Farm (Zhejiang, China). The animals were anesthetized intravenously with 50 mg/mL Sutent 50 (0.1 mL/kg). A corneal trephine was used to create four circular wounds, each with a 10 mm diameter, on the ventral aspect of each ear, with a minimum spacing of 10 mm between the wounds. After a 3-week period for wound healing and scar maturation, the hydrogels were applied to the scars. The laser exposure group received laser irradiation (L-2, 660 nm, 0.3 W/cm^2^, 5 min) once weekly, and scar morphology was monitored *via* photography. After 8 weeks, scar tissues were collected and either fixed in PFA for histology or stored at -80 °C for molecular analysis. Histological analyses were conducted on the fixed tissues via H&E staining to assess tissue morphology, Masson's trichrome staining for collagen deposition, Sirius Red staining under polarized light to evaluate collagen fiber organization, and TUNEL immunofluorescence for apoptotic cell detection. Scar tissue stored at -80 °C was used for sequencing, which was conducted on the Illumina NovaSeq 6000 platform. DEGs were identified *via* DESeq2 software with the criteria of |log_2_-fold change| ≥ 0.585 and Q ≤ 0.05. GO enrichment analysis and KEGG pathway enrichment analysis were subsequently performed for bioinformatics evaluation.

### Statistical analysis

GraphPad Prism 10.0 software (Dotmatics, MA, USA) was used for graph creation and statistical analysis. The data are presented as the means ± standard deviations (SDs). One-way analysis of variance (ANOVA) was employed for statistical evaluation. The results were considered statistically significant if the *P* value was less than 0.05. Significant differences are marked with symbols.

## Results and Discussion

### Synthesis and characterization of PCCUR

**Figure 2:**
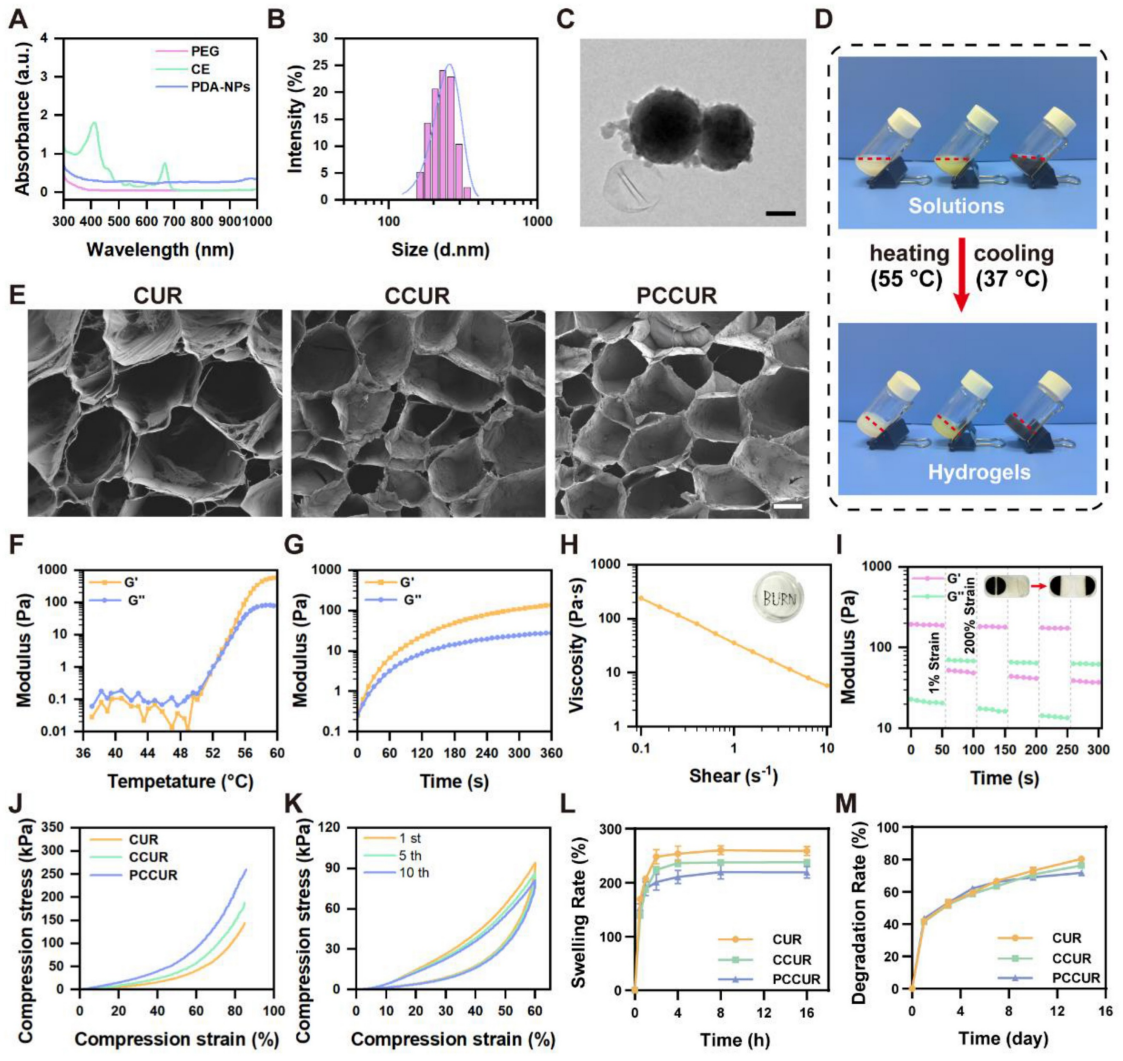
** Fabrication and characterization of PCCUR.** (A) UV‒Vis absorption spectra of the PEG, CE, and PDA-NPs. (B) Particle size distribution and (C) TEM images of PDA-NPs (scale bars = 100 nm). (D) Representative optical image of the fabricated hydrogels. (E) SEM images of the various hydrogel formulations (scale bars: 100 μm). (F) Temperature-dependent rheological properties of PCCUR. (G) Rheological analysis of PCCUR in time mode at a frequency of 10 Hz. (H) Viscosity (Pa·s) of the PCCUR hydrogel as a function of shear rate (s^-1^). (I) Storage modulus (G') and loss modulus (G'') of the PCCUR hydrogel as assessed by shear strain (1-200%) for 3 cycles. Photographs of the self-healing properties of the hydrogels: CUR (white) and PCCUR (black). (J) Compression stress‒strain curves of the hydrogels. (K) Mechanical recovery stability after 10 consecutive loading‒unloading compression cycles at 60% strain. (L) Real-time swelling ratio of the hydrogels. (M) Degradation rates of the hydrogels.

Following established protocols, PEG was employed as a solvent to extract active ingredients from wall-broken *Chlorella* powder, yielding CE, which had a chlorophyll content of 702.3 μg/mL. UV‒Vis spectroscopy analysis of the CE revealed distinct absorption maxima at 410 nm and 660 nm (**Figure 2A**), confirming the presence of chlorophyll 50. A 10% volume fraction of CE was selected for subsequent hydrogel preparation *via* the HFBs cytocompatibility test (**Figure S1**). PDA-NPs were synthesized from dopamine in an alkaline solution following a previously described method 53. UV‒Vis absorption spectra revealed that the PDA-NPs exhibited absorption across a broad wavelength range of 400-1000 nm, indicating their ability to absorb light in the ultraviolet to visible spectrum (**Figure 2A**). The synthesized PDA-NPs were characterized *using* dynamic light scattering (DLS) and transmission electron microscopy (TEM), which revealed a uniform spherical morphology with an average diameter of 255.73 ± 3.29 nm (**Figure 2B, C**).

PCCUR was created by introducing PDA-NPs and curdlan powder into a diluted CE solution, followed by heat induction to induce sol‒gel transition, as described previously 54. The CCUR hydrogel contains both CE and curdlan, whereas the CUR hydrogel contains only curdlan (**Figure 2D**). Curdlan serves as the primary structural framework of the hydrogel, with its thermoresponsive properties governing the fundamental mechanism of sol‒gel transition. Upon heating, curdlan molecules undergo a conformational change from disordered single helices to ordered triple helices. During the cooling process, these triple helical structures form a three-dimensional physical network through intermolecular hydrogen bonding and hydrophobic interactions 55-57. We utilized FTIR to analyze the characteristic absorption peaks of CUR powder, CE, PDA-NPs, and three types of hydrogels (CUR, CCUR, and PCCUR) in detail. As shown in **Figure S2**, the characteristic absorption peaks of the CUR powder are predominantly located at 3443 and 1647 cm^-1^ (O-H stretching vibration), 2920 and 1378 cm^-1^ (C-H), and 1078 cm^-1^ (C-O-C). The CE exhibited characteristic absorption peaks at 3406 and 1650 cm^-1^ (O-H), 2926 and 1404 cm^-1^ (C-H), and 1030 cm^-1^ (C-O-C). The characteristic absorption peaks of the PDA-NPs are found at 3444 cm^-1^ (N-H) and 1626 cm^-1^ (O-H). Compared with those of the CUR powder, CE, and PDA-NPs, the absorption peaks of O-H (approximately 3400 and 1650 cm^-1^), N-H (approximately 3444 cm^-1^), and C-H (approximately 2900 and 1400 cm^-1^) were significantly lower. This may be attributed to the formation of hydrogen bonds between these groups, which promote intermolecular cross-linking and construct a stable hydrogel network. Moreover, the absorption peaks of the three hydrogels near 1024 cm^-1^ (C-O-C) and 887 cm^-1^ (C-C) also decreased, indicating that as the degree of cross-linking increased, the vibration of these chemical bonds was restricted, leading to weakening of the corresponding infrared absorption peaks. This trend of enhanced cross-linking is most pronounced in PCCUR, suggesting that the addition of PDA further facilitates the formation and stabilization of the hydrogel network. These results demonstrate that the incorporation of CE and PDA-NPs can effectively enhance the degree of cross-linking of CUR-based hydrogels. Furthermore, we scaled up the preparation of PCCUR, as illustrated in **Figure S3A**, demonstrating that an increase in preparation volume from 500 μL to 60 mL did not compromise the gelation properties of PCCUR. SEM confirmed the formation of a three-dimensional porous network structure in all the hydrogels (**Figure 2E**). The microarchitecture of these hydrogels could facilitate the retention of CE and PDA-NPs, increase gas exchange, and efficiently absorb tissue exudate, all of which are crucial for postoperative melanoma treatment. As depicted in **Figure S3B**, the average pore size of the PCCUR hydrogel is approximately 187.33 μm, which is conducive to cellular migration and adequate spreading 58. The thermal sensitivity of PCCUR was evaluated *via* rheometry. As shown in **Figure 2F**, the storage modulus (G′) and loss modulus (G″) of PCCUR significantly increased at approximately 51 °C, with G′ surpassing G″, indicating a sol‒gel transition in the solution. Beyond this point, G′ remained significantly greater than G″ over the entire temperature range examined, demonstrating the stable gel state of PCCUR. Furthermore, under isothermal conditions at 55 °C, time-dependent changes in the G'/G" ratio were observed, with a peak reached at 5 min (**Figure 2G**). This phenomenon is consistent with the literature on the thermosensitivity of CUR, and the addition of CE and PDA-NPs did not alter its inherent properties 59, 60. We subsequently evaluated the injectability of PCCUR through shear thinning experiments. The results indicated that the viscosity of PCCUR decreased by two orders of magnitude with increasing angular frequency, demonstrating its shear-thinning properties (**Figure 2H**). This characteristic indicates that PCCUR is highly injectable, making it well suited for application as a wound dressing. To further assess its self-healing ability, rheological analysis was performed under alternating high- and low-strain conditions. **Figure 2I** shows a sharp decline in the G' of PCCUR under high strain (200%). However, after three-step strain cycles, both G' and G'' rapidly recovered at a low strain of 1%, indicating that PCCUR exhibited remarkable self-healing capabilities.

Typically, ideal hydrogel dressings should have excellent mechanical properties to protect wounds from further damage 61, 62. Therefore, we conducted mechanical performance tests on the hydrogels. As shown in **Figure 2J and Figure S4A**, the hydrogels demonstrated excellent elasticity under compression, with all three hydrogels able to withstand 80% compressive strain without significant rupture. Notably, PCCUR exhibited the highest compressive strength, reaching 261.06 kPa, indicating that PDA-NPs incorporation significantly improved the mechanical properties of the hydrogels. This mechanical enhancement may result from the hydrogen bonding network formed between PDA's phenolic hydroxyl groups and curdlan's hydroxyl groups, which provides additional crosslinking points, consequently reinforcing the structural integrity of the hydrogel 63. Furthermore, we subjected the PCCUR samples to evaluate their stability under repetitive compression. After 10 compression‒recovery cycles at 60% strain, the compressive strength decreased by only 13%, indicating strong resistance to compressive stress and excellent shape recovery (**Figure 2K**). The adhesive performance was evaluated *via* a lap shear test to simulate the interaction between the hydrogel and biological tissues. As shown in **Figure S4B**, the maximum adhesive strength of PCCUR to porcine skin reached 17 kPa. Collectively, these mechanical tests indicate that PCCUR possesses superior compressive properties and adhesive capabilities, suggesting its potential for stable application in skin wounds.

Swelling properties are critical for reducing wound exudate and lowering the risk of infection 64. Excessive hydrogel swelling causes burst release and a significant reduction in mechanical properties, which is detrimental to sustained wound protection 65. **Figure 2L** shows that all the hydrogels rapidly absorbed water within the initial 4 h. By 16 h, the swelling rates of the CUR, CCUR, and PCCUR hydrogels were 259.01 ± 8.02%, 238.32 ± 4.91%, and 219.42 ± 10.77%, respectively. Further *in vitro* degradation experiments, depicted in **Figure 2M**, revealed that all the hydrogels had similar degradation profiles. The CUR hydrogel had a slightly faster degradation rate, reaching 80.35 ± 1.77% over 14 days, whereas the CCUR and PCCUR hydrogels had slower degradation rates (76.35 ± 1.81% and 71.73 ± 1.10%, respectively). This slight reduction in the swelling and degradation profiles of PCCUR may be due to the incorporation of CEs and PDA-NPs, which may endow the hydrogel with increased structural rigidity. The release profiles of PCCUR are presented in **Figure S5**. PCCUR provided sustained release of both the CE and the PDA-NPs over 14 days. The cumulative release rates on day 14 were 84.01 ± 1.71% for the PDA-NPs and 98.27 ± 0.76% for the CE, indicating that the more densely cross-linked network hinders passive diffusion, thereby prolonging the release of the CE and PDA-NPs. This extended release profile allows for sustained photothermal and photodynamic effects, enabling synergistic therapy. Comparative analysis revealed that, compared with CE, PDA-NPs presented greater cumulative release and faster release kinetics. This enhanced release performance is likely attributable to the superior hydrophilicity of the PDA-NPs, facilitating more rapid diffusion from the hydrogel matrix.

Therefore, the synthesized PCCUR exemplifies an advanced fabrication strategy, seamlessly incorporating PDA-NPs, CE, and CUR within a thermally driven, physically cross-linked matrix. This design not only bolsters the hydrogel's mechanical resilience but also introduces essential functionalities, including injectability, self-healing, and adhesive properties. As highlighted in previous studies, such attributes are pivotal for maximizing the therapeutic applications of hydrogels 48, 66. Additionally, PCCUR is uniform and has a reduced pore size, increasing its structural coherence and mitigating the uneven swelling often associated with conventional hydrogels. Its injectability also makes it suitable for filling irregular postoperative wounds of melanoma. The hydrogel's precisely tunable swelling and degradation behaviors facilitate the sustained release of therapeutic factors and foster an optimal wound-healing microenvironment, thereby minimizing postoperative complications 67. PDA further strengthens the hydrogel by enhancing polymer chain interactions, promoting both entanglement and cross-linking within the curdlan matrix, resulting in customizable mechanical properties 68.

### Evaluation of laser responsiveness and biocompatibility of PCCUR hydrogels

**Figure 3:**
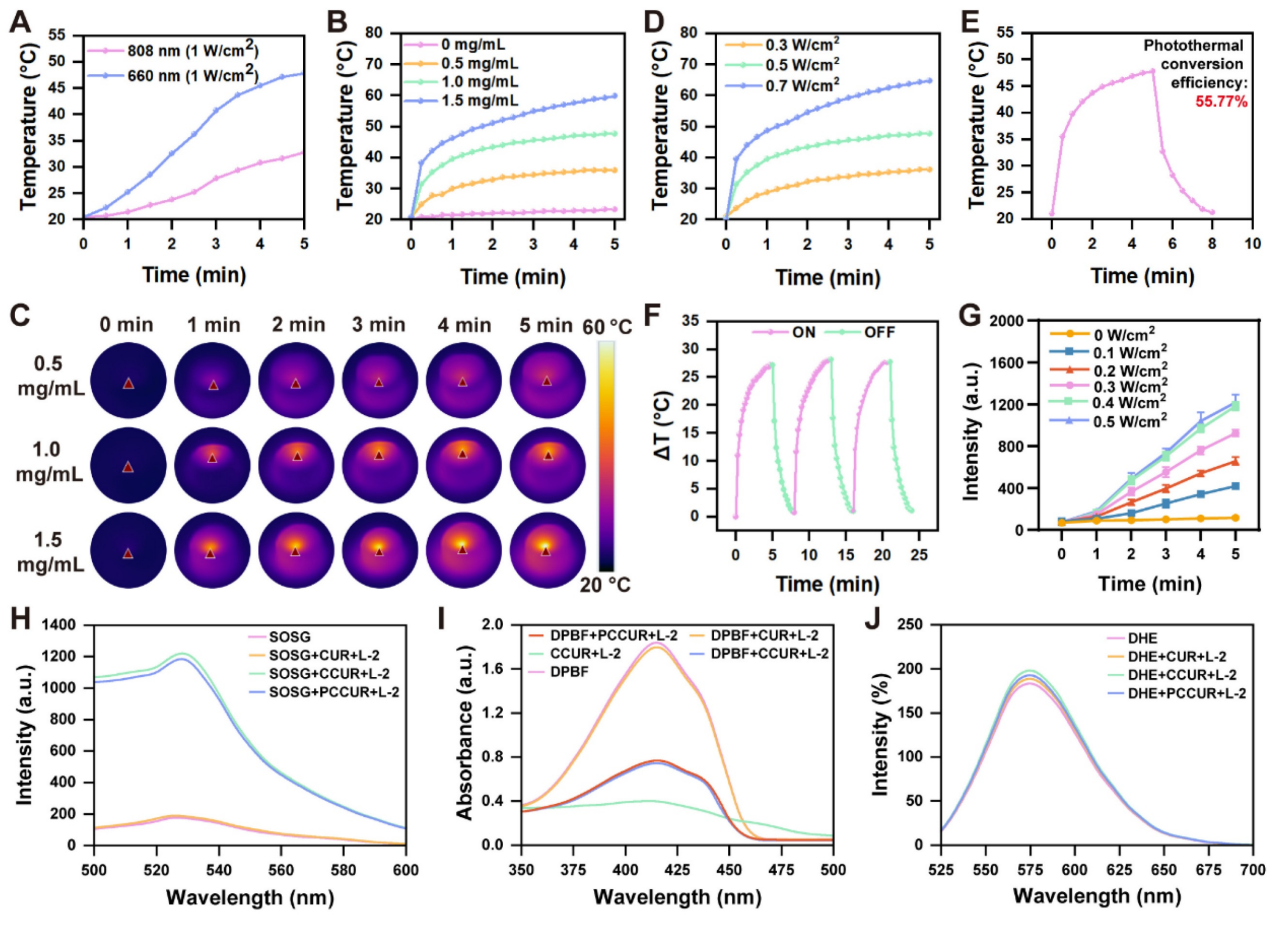
** Evaluation of PCCUR photoresponse under laser irradiation.** (A) Temperature profiles of a 1 mg/mL PDA-NPs solution irradiated with 808 nm and 660 nm lasers at the same power (1 W/cm^2^). (B) Temperature profiles and (C) photothermal images of PCCUR hydrogels containing varying PDA-NPs concentrations under laser irradiation (0.5 W/cm^2^, 5 min). (D) Temperature variation curves of PCCUR subjected to laser irradiation at various power intensities. (E) Heating profile of PCCUR with a 660 nm laser (0.5 W/cm^2^) and cooling profile after laser shutdown. (F) Temperature variation of PCCUR (1 mg/mL PDA-NPs) over three laser irradiation on/off cycles using a 660 nm laser at a power density of 0.5 W/cm^2^. (G) Fluorescence intensity of SOSG as a function of irradiation time at different laser irradiation powers. (H) SOSG fluorescence intensity and (I) DPBF absorbance measurements confirmed significant ¹O₂ production in PCCUR hydrogels under 660 nm laser irradiation (0.3 W/cm², L-2). (J) DHE fluorescence intensity showing no significant superoxide anion (O₂⁻˙) production across groups. The data are presented as the means ± SDs (n = 3).

The 808 nm laser has traditionally served as the main option for photothermal conversion in PDA-NPs 69, 70. Notably, our observations indicated that compared with 808 nm laser irradiation, exposure of PDA-NPs to a 660 nm laser at the same power level significantly increased their temperature (**Figure 3A**). To further investigate the photothermal effects, we examined PCCUR loaded with various concentrations of PDA-NPs under a 660 nm laser (0.5 W/cm^2^) *via* an infrared thermal imaging camera. **Figure 3B-D** presents the temperature variation curves and photothermal images of the hydrogels under different conditions. After 300 s of laser irradiation, the CCUR showed no noticeable temperature increase. In contrast, the temperature of PCCUR loaded with 1 mg and 1.5 mg of PDA-NPs increased to 47.7 °C and 59.8 °C, respectively, which was significantly higher than that of PCCUR loaded with 0.5 mg of PDA-NPs (35.8 °C). Tumor cells are quickly eliminated by microvascular thrombosis and ischemia at temperatures ranging from 46 °C to 52 °C. When tissue temperatures surpass 60 °C, immediate cell death occurs because of protein denaturation and disruption of the plasma membrane 71. PTT has been increasingly utilized at temperatures under 50 °C and with exposure durations of less than 10 min 72, 73. Next, we assessed the heating effect of PCCUR loaded with 1 mg of PDA-NPs at different power levels. The results indicated no significant temperature increase at 0.3 W/cm^2^ (36.1 °C), whereas the temperature was excessively high at 0.7 W/cm^2^ (64.8 °C). Therefore, a power level of 0.5 W/cm^2^ was deemed optimal. Furthermore, the photothermal conversion efficiency of the PCCUR hydrogel, as deduced from **Figure 3E**, reached 55.77%, markedly surpassing that of other PDA-based nanomaterials, including NRs@PDA (40.18%) 74, cyclodextrin-functionalized PDA-Pt (44.5%) 75, and ultrathin MnO2@PDA (28.75%) 76. After three cycles of laser irradiation (0.5 W/cm^2^), PCCUR exhibited minimal thermal fatigue loss, indicating excellent photostability (**Figure 3F**). To explore the ROS generation capability of PCCUR under PDT, we employed a singlet oxygen sensor green (SOSG) fluorescence probe in conjunction with a fluorescence spectrometer. PCCUR was incubated with the SOSG probe solution and subsequently subjected to laser irradiation at different power densities. As shown in **Figure 3G**, the fluorescence intensity of SOSG gradually increased over 5 min with intensified 660 nm laser irradiation, indicating that PCCUR effectively triggered ROS production upon laser exposure. Therefore, PCCUR exhibited efficient thermal elevation and ROS generation capacity upon exposure to a 660 nm (0.5 W/cm^2^) laser, which is a critical prerequisite for synergistic PTT/PDT therapy against tumors and infections 77, 78. To further evaluate the ROS generation capacity of hydrogels under 660 nm (0.3 W/cm², L-2) laser irradiation, we employed three fluorescent probes: SOSG, DPBF, and DHE. As shown in **Figure 3H**, compared with the untreated group, no significant change in fluorescence intensity was observed in the CUR+ L-2 group, while a significant increase in fluorescence intensity was noted in both the CCUR+ L-2 and PCCUR+ L-2 groups. This suggests that the incorporation of CE endows the hydrogel with the ability to mediate the generation of ^1^O_2_ during PDT. Additionally, the fluorescence intensity in the PCCUR+ L-2 group was slightly lower than that in the CCUR+ L-2 group. We attribute this minor attenuation to the competitive quenching of a minimal fraction of ¹O₂ by PDA-NPs, while the majority of ^1^O_2_ remains bioavailable. We subsequently employed the DPBF probe to further validate the aforementioned results. The absorbance in the CUR+L-2 group was comparable to that in the untreated group, whereas a significant decrease in absorbance was observed in both the CCUR+L-2 and PCCUR+L-2 groups (**Figure 3I**). To determine the production of other types of ROS, DHE was employed to detect O_2_·. No significant differences in fluorescence intensity were observed among the untreated, CUR, CCUR, and PCCUR groups under L-2 laser irradiation, indicating no measurable O_2_· production (**Figure 3J**). Collectively, these results demonstrate that the PCCUR hydrogel irradiated at 660 nm (0.3 W/cm²) mediates photodynamic effects via targeted ¹O₂ generation.

Hemolysis assays were carried out to determine the biocompatibility of the hydrogels. Visual inspection revealed that the positive control group contained red supernatant, whereas the three hydrogel groups and the negative control (PBS) maintained clear supernatants, confirming their high hemocompatibility (**Figure S3**). Compared with those of CUR, the hemolytic rates of the PCCUR and CCUR hydrogels increased, potentially due to the absorbance of the CE and PDA-NPs at 545 nm. *In vivo* biosafety was assessed by subcutaneously implanting the hydrogels into the dorsal area of mice. As shown in **Figure S7**, all three hydrogels underwent gradual degradation over time. H&E staining revealed a distinct boundary between the hydrogels and surrounding tissue on day 3. By day 7, this boundary had become blurred. By day 10, the CUR hydrogels had almost completely degraded, with a marked reduction observed in the CCUR and PCCUR groups. Complete degradation of all the hydrogels was achieved by day 14, attesting to their robust biodegradability and biocompatibility* in vivo*. The faster degradation rate *in vivo* compared with *in vitro* PBS immersion is principally ascribed to the enzymatic activity and cellular clearance mechanisms inherent to the biological environment, which accelerate breakdown beyond the hydrolytic processes that are dominant in static PBS 79, 80. Fourteen days postimplantation, the analyses demonstrated that routine blood and liver/kidney function tests for both the hydrogel and control groups remained within normal ranges (**Figure S8**). H&E staining of major organs, including the heart, liver, spleen, lungs, and kidneys, did not reveal any significant pathological changes, confirming the safety of the treatments (**Figure S9**).

### *In vitro* cellular effects of PCCUR

**Figure 4:**
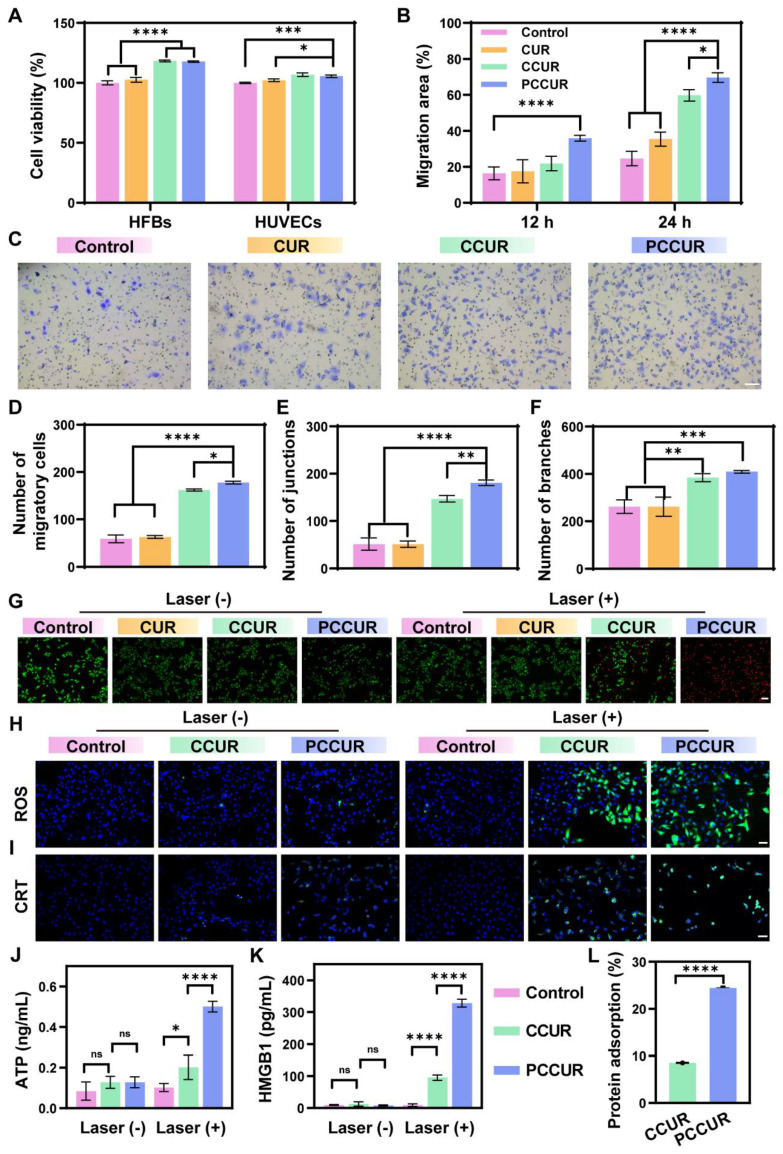
***In vitro* cellular effects of PCCUR.** (A) Viability of HFBs, HUVECs, and HaCaT cells treated with different hydrogels for 24 h. (B) Quantification of the migration area of HFBs at different time points posttreatment. (C) Representative images and (D) quantification of the Transwell migration of HaCaT cells (scale bars: 100 μm). Quantification of (E) junction formation and (F) branch formation in the HUVECs tube formation assay. (G) Live/dead assay of B16F10 cells following various treatments (scale bars: 100 μm). (H) Detection of ROS in B16F10 cells after different treatments *via* the fluorescent probe DCFH-DA. (I) Confocal microscopy images showing CRT (green) exposure in B16F10 cells after different treatments (scale bars: 50 μm). Amounts of (J) ATP and (K) HMGB1 released from B16F10 cells under different treatments. (L) Protein adsorption in FBS by various hydrogels. The data are presented as the means ± SDs (n = 3). ns (not significant), *P < 0.05, **P < 0.01, ***P < 0.001, ****P < 0.0001. Laser (660 nm, 0.5 W/cm^2^).

The presence of pathogens, ischemia, and oxidative stress in infected wounds impedes the movement and survival of skin cells. 81-83. Therefore, wound dressings must support cell proliferation, migration, and angiogenesis 84. HFBs and HUVECs are integral to wound healing 85. *In vitro* studies assessed the impact of CUR, CCUR, and PCCUR hydrogels on cell proliferation *via* CCK-8 assays, which demonstrated good cytocompatibility of HFBs upon treatment with CUR, CCUR, or PCCUR. Among these, PCCUR and CCUR both significantly induced the proliferation of HFBs (**Figure 4A**). A comparable trend was shown in HUVECs. These findings indicate that PCCUR could significantly increase cell proliferation in wound tissues. To assess the migratory potential of HFBs and HUVECs, we performed scratch wound healing and Transwell migration assays. Compared with the control, PCCUR significantly increased the migratory capacity of both HFBs and HUVECs (**Figure 4B-D and S10**).

Angiogenesis, the development of new blood vessels, involves endothelial cells growing, moving, and forming tubes 86. This process is essential for the healing of wounds, as the newly created vessels supply the affected area with oxygen and nutrients 87. To examine the impact of CUR, CCUR, and PCCUR hydrogels on angiogenesis *in vitro*, we performed tube formation assays. The CCUR and PCCUR groups exhibited a significantly greater numbers of vessel junctions and branches in HUVECs compared to the control group, indicating strong induction of angiogenesis (**Figure 4E-F and S11**). Collectively, our results confirmed that PCCUR effectively promoted wound healing by increasing cell proliferation, migration, and tubulogenesis.

The* in vitro* antitumor efficacy of PCCUR was determined by assessing B16F10 cell viability via CCK-8 assays and live/dead staining. As shown in **Figure S12**, in the absence of laser irradiation, the cell viability remained above 90% in the presence of all three hydrogel cultures, indicating their excellent biocompatibility. After exposure to laser irradiation, there was a marked reduction in cell viability. Specifically, the groups treated with CCUR and PCCUR presented a significant decrease in viability, with mean values of 51.58% ± 0.54% and 22.88% ± 0.76%, respectively. These results suggest that the PCCUR treatment was more effective in reducing cell viability compared to the CCUR treatment. These results were further confirmed by live/dead staining (**Figure 4G**). In the groups devoid of laser irradiation, minimal cell mortality was observed. The PCCUR + laser group presented the most intense red fluorescence, indicating a pronounced induction of cell death. In contrast, the CCUR + laser group demonstrated a moderate degree of cell mortality. Notably, the CUR group displayed no significant difference in cell viability under either laser or nonlaser conditions. Given that CUR alone has no substantial antitumor activity, it was excluded from further experimental controls. To assess the photoinduced generation of ROS within B16F10 cells more rigorously, we employed 2',7'-dichlorodihydrofluorescein diacetate (DCFH-DA). As depicted in **Figure 4H**, the CCUR + laser group exhibited robust green fluorescence indicative of ROS, suggesting that the chlorophyll in the CE can generate considerable amounts of ROS under laser irradiation. Most intriguingly, the PCCUR + laser group presented the most significant elevation in intracellular ROS levels, implying that the thermal elevation caused by PDA-NPs could augment the PDT efficiency of CE.

PDT and PTT have been demonstrated to induce ICD in tumor cells, which enhances tumor immunogenicity by promoting the release of tumor-associated antigens (TAAs) 88. In the ICD process, several damage-associated molecular patterns (DAMPs), such as cell surface CRT, HMGB1, and ATP, play crucial roles in the activation of the immune response, including the recruitment of DCs and the activation of T cells 89. Confocal laser scanning microscopy (CLSM) was employed to evaluate the exposure of CRT on the cell surface. As depicted in **Figure 4I**, cells subjected to PCCUR or CCUR treatments presented minimal CRT exposure. In stark contrast, cells treated with PCCUR in conjunction with laser irradiation displayed increased CRT fluorescence, indicative of efficient ICD induced by the synergistic effects of PTT and PDT. To further substantiate the induction of ICD, we quantified the levels of ATP and HMGB1, which are canonical biomarkers of ICD. The ATP levels in the CCUR + laser and PCCUR + laser groups were elevated by 2.38-fold and 5.91-fold, respectively, compared with those in the control group (**Figure 4J**). Similarly, the HMGB1 concentrations in the CCUR + laser and PCCUR + laser groups were markedly greater, being 10.19-fold and 35.08-fold greater than those in the control group, respectively (**Figure 4K**). Collectively, these results underscore the potent induction of ICD by PCCUR + laser treatment, which is mediated through the release of various DAMPs *via* the combined modality of PDT and PTT. Considering the capacity of the catechol groups in the PDA-NPs to adsorb antigens, we assessed the antigen adsorption ability of the PCCUR hydrogel. As shown in **Figure 4L**, the protein adsorption capacity of the PCCUR hydrogel in fetal bovine serum (FBS) was nearly threefold greater than that of the CCUR hydrogel.

CE is renowned for its abundant bioactive constituents, which have been shown to stimulate the proliferation of diverse cell lineages 90. Our findings indicate that CE has profound proliferation-promoting effects on three distinct skin cell types, underscoring its exceptional biocompatibility. This effect is attributed to the presence of polysaccharides, polypeptides, and other nutrients within the CE, which actively contribute to cellular growth. Furthermore, the biocompatibility of curdlan-based hydrogels has been previously established 56, 91. PDA-NPs are known to augment cell migration, with their therapeutic potency attributed to functional groups such as catechol and quinone 92. The amalgamation of CE with PDA-NPs within PCCUR is hypothesized to elicit a synergistic effect, thereby enhancing the proliferation, migration, and tubulogenesis of skin cells. Upon laser irradiation, PCCUR plays a dual role: it acts as a direct antitumor agent through the synergistic application of PDT and PTT, and it functions as an adjuvant to bolster the antitumor immune response *via* phototherapy-induced ICD. Moreover, PCCUR may also act as an "antigen reservoir." As demonstrated by Fan *et al.*, adhesive hydrogels can capture tumor-derived protein antigens released during PTT, thereby acting as “antigen reservoirs” to amplify and sustain immune stimulation 42. Consequently, the direct cytotoxic effects, induced ICD, and antigen adsorption capacity of PCCUR are posited to confer potent antitumor effects *in vitro*. In conclusion, PCCUR embodies a holistic therapeutic strategy that integrates wound repair and antitumor effects by modulating laser exposure during specific treatment phases. Our study revealed that PCCUR not only has an exceptional capacity to promote skin cell proliferation, migration, and tubulogenesis but also exerts significant antitumor effects. These findings underscore the potential of PCCUR in the comprehensive management of postsurgical melanoma.

### *In vitro* antibacterial activities of PCCUR

**Figure 5:**
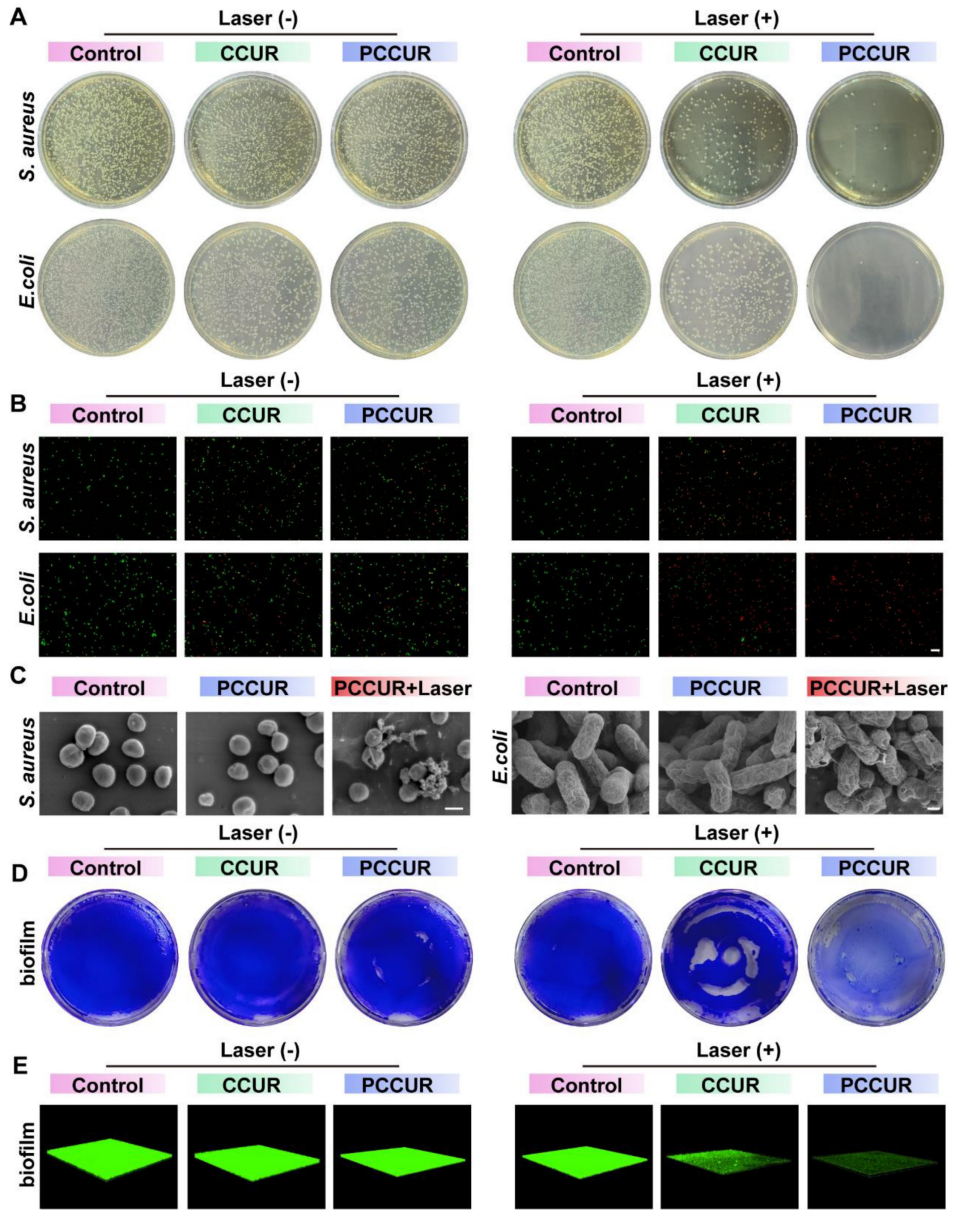
***In vitro* antibacterial activity of PCCUR *via* PDT-PTT synergy.** (A) Representative plate images showing colony formation of *S. aureus* and *E. coli* following different treatments. (B) Fluorescence microscopy images of *S. aureus* and *E. coli* stained with SYTO 9/PI posttreatment (scale bars: 20 μm). (C) Representative SEM images of *S. aureus* (scale bars: 500 nm) and *E. coli* (scale bars: 300 nm). (D) Photographs of stained MRSA biofilms following different treatment protocols. (E) Three-dimensional confocal laser scanning microscopy (CLSM) images of preestablished MRSA biofilms following different treatments. Laser (660 nm, 0.5 W/cm^2^).

The complex wound microenvironment following tumor removal is prone to bacterial colonization, requiring prompt antimicrobial treatment during the inflammatory phase to prevent chronic, nonhealing wounds 93. To evaluate the *in vitro* antibacterial capabilities of PCCUR, we selected two prevalent pathogens: *Staphylococcus aureus* (*S. aureus*) and *Escherichia coli* (*E. coli*). In the absence of laser irradiation, the antibacterial rates of the CCUR and PCCUR groups against *S. aureus* were 13.79 ± 1.64% and 16.29 ± 0.34%, respectively, whereas against *E. coli*, the rates were 48.74 ± 0.82% and 54.85 ± 4.87%, respectively (**Figure 5A** and **Figure S12A-B**). These results suggest that both CCUR and PCCUR exhibit moderate antibacterial effects against *S. aureus* and *E. coli* without laser irradiation. Upon laser irradiation, the antibacterial rates against *S. aureus* significantly increased to 81.91 ± 5.09% and 97.67 ± 1.89% for CCUR and PCCUR, respectively. Similarly, the antibacterial efficacy against *E. coli* increased to 74.29 ± 3.36% and 99.55 ± 0.13% for the CCUR and PCCUR groups, respectively. Compared with CCUR, PCCUR had a more pronounced inhibitory effect on *S. aureus* and *E. coli* under laser irradiation. These findings suggest that the PDT-mediated antibacterial properties of CCUR and PCCUR may be attributed to the CE. The incorporation of PDA-NPs into the hydrogel further enhanced the photodynamic antibacterial effect of CCUR, enabling PCCUR to effectively inhibit *S. aureus* and *E. coli* through synergistic photothermal and photodynamic effects.

To address the complex wound microenvironment after tumor excision, which is prone to bacterial colonization, we focused on the antibacterial properties of PCCUR. Live/dead bacterial staining was conducted to determine the antibacterial efficacy of the hydrogels. As depicted in **Figure 5B**, the laser-irradiated PCCUR group alone displayed intense red fluorescence for both *S. aureus* and *E. coli*, indicating a substantial number of bacterial deaths. To further evaluate the bactericidal potency of PCCUR, SEM was employed to examine bacterial morphology and integrity (**Figure 5C**). Without laser irradiation, PCCUR-treated *S. aureus* and *E. coli* exhibited slight surface wrinkling compared to the control group. However, upon laser irradiation, both bacterial species demonstrated severe deformation, shrinkage, and even rupture. These effects are believed to result from the combined action of the ROS generated by PDT and localized hyperthermia induced by PTT.

Biofilm formation complicates bacterial infections by increasing resistance to treatment 94, 95. To assess this, we stained methicillin-resistant *S. aureus* (MRSA) biofilms with crystal violet and evaluated their biomass posttreatment (**Figure 5D** and **Figure S13C**). The CCUR + laser group showed partial biofilm disruption, whereas the PCCUR + laser group exhibited complete destruction, highlighting its potent antibacterial activity. In contrast, biofilms in the other groups retained their vitality and structure. Three-dimensional reconstruction of MRSA biofilms *via* CLSM further visualized biofilm disruption (**Figure 5E**). The control group displayed intact biofilms with strong green fluorescence, whereas the PCCUR + laser group showed significant biofilm inhibition. These findings highlight the potency of the integrated PTT and PDT approach in inhibiting bacterial growth and disrupting biofilms, which are often refractory to conventional therapies 96, 97.

This study introduces PCCUR as an innovative antibacterial matrix, leveraging the synergistic mechanisms of PTT and PDT to combat postsurgical infections following melanoma excision. PCCUR strongly inhibited *S. aureus* and *E. coli* and efficiently disrupted MRSA biofilm formation under 660 nm laser irradiation. The antimicrobial efficacy of PDT is attributed to the activation of chlorophyll, which generates ^1^O^2^ and other ROS, inducing oxidative stress and compromising bacterial membranes 78, 98. However, the limited range and short lifespan of ROS reduce their overall efficacy. To overcome this, we employed a combined PDT/PTT strategy, where mild hyperthermia enhances membrane permeability, increasing bacterial susceptibility to oxidative damage and amplifying the antibacterial effect 99. Controlled laser application mitigates the risk of excessive ROS and heat production, reducing the risk of injury to healthy tissues 100. This dual-modal strategy, which enables simultaneous PTT and PDT activation *via* a single laser, simplifies the therapeutic procedure and represents a significant advancement over previous single-modality treatments, underscoring the innovative potential of our approach in managing infections associated with melanoma resection.

### *In vivo* antitumor assays

**Figure 6:**
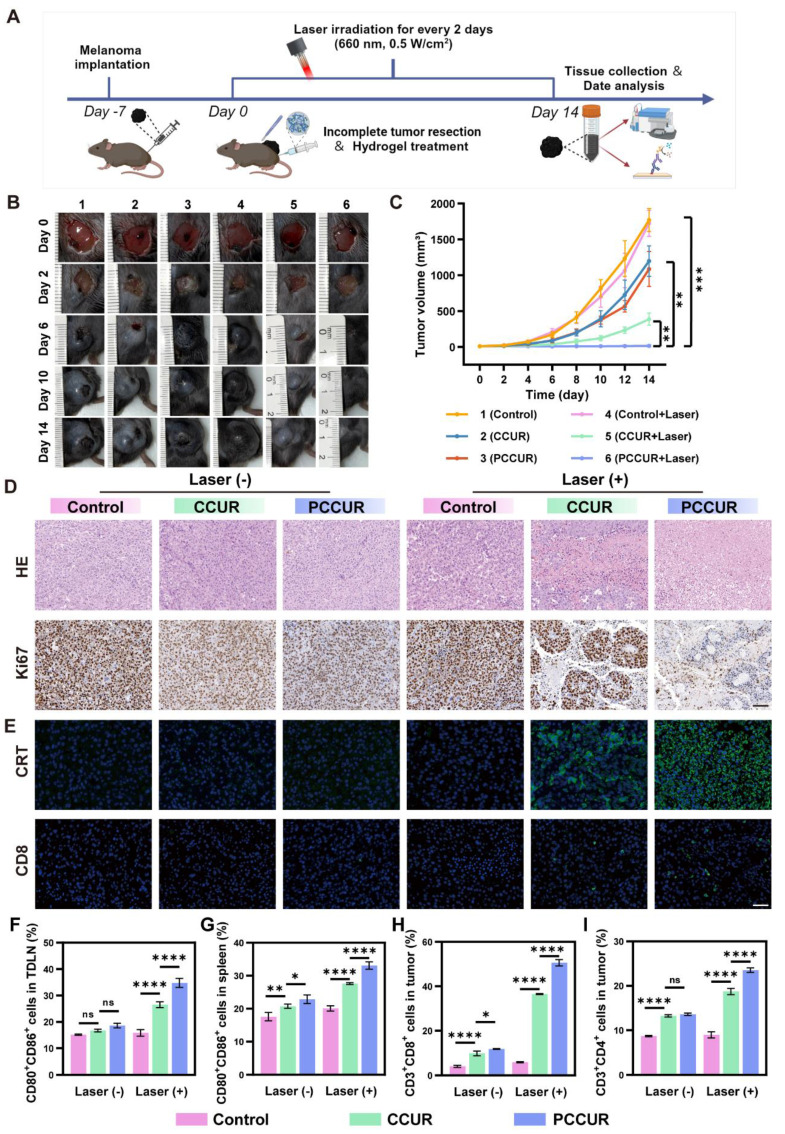
***In vivo* therapeutic effects of PCCUR on melanoma recurrence**. (A) Schematic representation of the incomplete melanoma resection model and the *in vivo* treatment protocols. Laser irradiation (660 nm, 0.5 W/cm^2^). (B) Photographs of tumor/wound sites in different groups during the 14-day treatment period. (C) Tumor recurrence volume curves over 14 days for each treatment group. (D) H&E and Ki67 immunohistochemical staining images of dissected tumor tissues (scale bars: 100 μm). (E) CRT and CD8 immunofluorescence staining of tumor tissue sections (scale bars: 50 μm). Quantitative analysis of mature DCs in (F) TDLNs and the (G) spleen by flow cytometry. Quantitative flow analysis results showing the presence of (H) CD3⁺CD8⁺ T cells and (I) CD3⁺CD4⁺ T cells in tumors. The data are presented as the means ± SDs (n =3 or 5). ns (not significant), *P < 0.05, **P < 0.01, ***P < 0.001, ****P < 0.0001. Laser (660 nm, 0.5 W/cm^2^).

To investigate the ability of PCCUR to inhibit tumor growth, we successfully established an incomplete resection melanoma model in C57BL/6 mice and administered different intervention treatments (**Figure 6A**). As shown in **Figure 6B**, the wounds in the PCCUR + laser group healed on postoperative day 14, with no tumor recurrence. In contrast, tumor recurrence was clearly observed in the other groups on postoperative day 6, with gradual tumor enlargement and even the development of ulcers. Tumor growth curves and final tumor weights were recorded for each treatment group after surgery (**Figure 6C, S14, and S15A**). Compared with the control group, the CCUR + laser group effectively inhibited tumor growth, with an inhibition rate of 78.27 ± 4.84%. These findings indicate that CCUR-mediated PDT could effectively ablate tumors. Notably, the PCCUR + laser group exhibited the most potent antitumor effect, achieving a tumor inhibition rate of 99.25 ± 0.78%, which highlights the synergistic efficacy of combined PDT and PTT in managing melanoma recurrence (**Figure S15B**). During the treatment period, there were no significant differences in body weight changes among the mice in all groups during the treatment period, suggesting that PCCUR exhibits low systemic toxicity (**Figure S16**).

Histopathological analyses of excised tumor tissues, including H&E staining and Ki-67 immunohistochemical staining, were performed (**Figure 6D**). The results revealed uncontrolled proliferation of tumor cells in the control group, whereas extensive tumor cell lysis was observed in the PCCUR + laser-treated group, as evidenced by the disappearance or dissolution of the nucleus and significantly reduced Ki-67 expression. Moreover, CRT immunofluorescence staining revealed that both the CCUR + laser and PCCUR + laser treatments induced robust CRT release, indicating the successful induction of ICD (**Figure 6E**). Similarly, CD8^+^ T-cell immunofluorescence revealed that PCCUR + laser treatment significantly increased the infiltration of CD8^+^ T cells in tumor tissue.

To investigate the mechanism driving the antitumor efficacy of PCCUR, we focused on immune cells present in the tumor microenvironment, specifically DCs and T cells, which are pivotal in initiating antitumor immunity, with their maturation serving as a key indicator of immune activation within the tumor microenvironment. Therefore, we evaluated the activation status of DCs in both the TDLNs and the spleen. Notably, PCCUR + laser treatment significantly enhanced DC maturation in both TDLNs and the spleen (**Figure S17A, Figure 6F-G**). The DC maturation rate in the PCCUR + laser group (34.77 ± 1.71%) was markedly greater than that in the CCUR + laser group (26.53 ± 1.12%), indicating that DCs are induced by the combination of PDT and PTT to activate immune function. Similar trends in DC maturation were observed in the spleen. To further evaluate the activation of T cells stimulated by antigen presentation by mature DCs, we extracted and examined T cells stimulated by antigens presented by mature DCs and isolated and analyzed T cells from tumor tissues (**Figure S17B, Figure 6H-I**). Compared with the other treatment groups, the PCCUR + laser treatment group presented the greatest number of infiltrating CD8^+^ T cells in the tumors (PCCUR + laser: 50.7 ± 1.56%, control: 4.77 ± 0.45%, *P* < 0.0001). Additionally, similar findings were observed for CD4^+^ T-cell infiltration in the tumor tissue. Taken together, these results showed that PCCUR not only induces ICD through the synergistic effect of PDT and PTT and enhances antigen cross-presentation but also triggers DC maturation, ultimately facilitating T-cell-mediated tumor eradication.

In this study, we delineated the efficacy of PCCUR in preventing tumor recurrence* via* an integrated approach encompassing PDT and PTT within an *in vivo* melanoma excision model. Our postexcision antirecurrence treatment paradigm more closely mirrors clinical scenarios than previous studies that predominantly targeted established tumors, thereby augmenting its clinical applicability and translational potential 23, 24, 101. In contrast to sequential therapy regimens, the single-laser methodology employed in this investigation concurrently induced PDT and PTT effects, thereby achieving significant antitumor efficacy and enhancing procedural simplicity 102, 103. The synergistic antitumor effects of PDT and PTT are attributed to distinct mechanisms. PDT induces the generation of ROS and cytotoxic free radicals, which directly trigger tumor cell death through apoptosis, necrosis, or autophagy 104, 105. In addition, PTT-induced hyperthermia increases cell membrane permeability, thereby amplifying the overall antitumor response 26. Furthermore, phototherapy instigates a robust and enduring antitumor immune response, including the induction of ICD, which liberates tumor-specific antigens and DAMPs, namely, CRT, HMGB1, and ATP 106, 107. The adhesive properties of the PCCUR hydrogel may augment therapeutic efficacy by functioning as an “antigen reservoir”, thereby preventing the premature clearance of these antigens by the innate immune system. The maturation of DCs and the infiltration of effector T lymphocytes suggest the initiation of a systemic immune response, which stimulates immune memory and fosters long-term antitumor immunity 108, 109. Our findings corroborate the burgeoning literature advocating the use of integrated phototherapy platforms as a potentially effective strategy against the recurrence of melanoma. The clinical translational potential of this strategy is considerable; however, future research should concentrate on optimizing treatment parameters and assessing safety and efficacy across a broader spectrum of cancer models. These forthcoming directions are pivotal for refining this innovative approach and ascertaining its feasibility in a variety of clinical settings.

### *In vivo* effects of PCCUR on infected wounds

**Figure 7:**
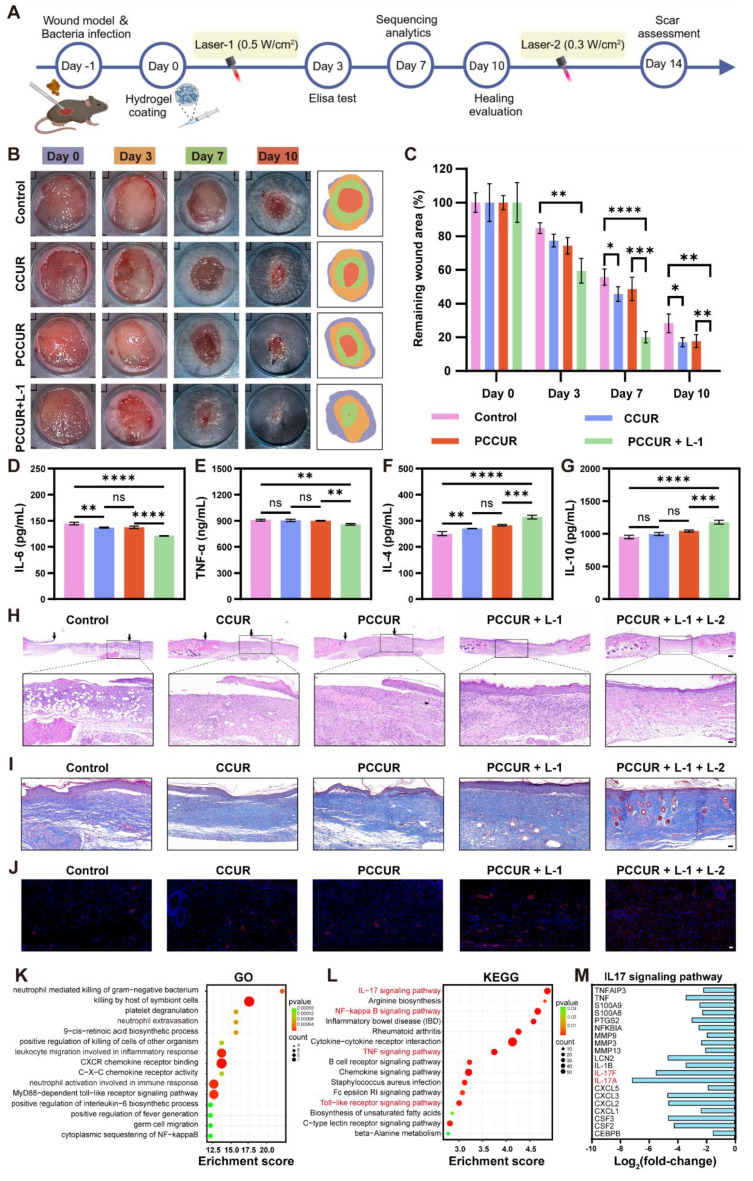
***In vivo* scarless healing of wounds.** (A) Schematic representation of the experimental procedures for treating infected wounds in mice with PCCUR combined with laser irradiation. (B) Representative photographs and simulation images depicting the wound healing process in mice. (C) Wound closure percentage at different time points. ELISA analysis of the expression levels of (D) IL-6, (E) TNF-α, (F) IL-4, and (G) IL-10 inflammatory factors in wound tissues on day 3. (H) H&E staining of wound tissues on day 10 posttreatment. Scale bar: 200 μm at the top and 50 μm in the enlarged images. (I) Masson's trichrome staining of wound tissues on day 14. Scale bar: 50 μm. (J) Immunofluorescence staining of α-SMA in wound tissues on day 14. Scale bar: 20 μm. (K) GO and (L) KEGG enrichment analyses of DEGs. (M) Changes in the expression levels of DEGs associated with the IL-17 signaling pathway following treatment with PCCUR + L-1. The data are presented as the means ± SDs (n = 5). ns (not significant), *P < 0.05, **P < 0.01, ***P < 0.001, ****P < 0.0001. Laser-1 (L-1, 660 nm, 0.5 W/cm^2^), Laser-2 (L-2, 660 nm, 0.3 W/cm^2^).

To assess the *in vivo* efficacy of PCCUR in promoting infected wound healing, we developed a full-thickness cutaneous lesion model in mice inoculated with *S. aureus* (**Figure 7A**). To address the varying needs of different wound healing stages, the laser power was adjusted accordingly throughout the 14-day treatment period. During the initial three days, a 660 nm laser at a power density of 0.5 W/cm² (Laser-1, L-1) was utilized to achieve antibacterial effects. For the last four days, the 660 nm laser power was reduced to 0.3 W/cm^2^ (Laser-2, L-2) for 5 min to inhibit scar proliferation. No laser irradiation was performed between days 4 and 10 postinjury. Wounds were photographed on days 0, 3, 7, and 10, and representative images are shown in **Figure 7B**. Quantitative analysis of the residual wound area (**Figure 7C**) indicated that, starting from day 7, the healing process in all three hydrogel groups, CCUR, PCCUR, and PCCUR + L - I, was more rapid than that in the control group. By day 10, the wounds in the PCCUR + L-I group were completely closed significantly faster than those in the control, CCUR, and PCCUR groups were, with remaining wound areas of 28.22 ± 5.59%, 17.07 ± 2.72%, and 17.78 ± 3.83%, respectively. Further analysis* via* ELISA revealed that on day 3, wounds treated with CCUR or PCCUR presented slight improvements in the levels of the proinflammatory cytokine interleukin-6 (IL-6) and the anti-inflammatory cytokine IL-4 compared with those in the control group, suggesting moderate anti-inflammatory effects of CCUR and PCCUR. However, wounds treated with PCCUR combined with laser irradiation presented significantly reduced levels of IL-6 and tumor necrosis factor-alpha (TNF-α) but markedly increased levels of the anti-inflammatory cytokines IL-4 and IL-10** (Figure 7D-G)**. In summary, these findings suggest that early application of high-energy laser irradiation with PCCUR, which combines PDT and PTT, efficiently alleviates inflammation and promotes accelerated wound healing.

Histological analysis was performed to evaluate the quality of the regenerated skin. By day 10, the PCCUR + L - 1 group uniquely formed a complete and continuous epidermis, whereas the other three groups exhibited discontinuous epidermises, with black arrows denoting the edges of the newly formed epidermis (**Figure 7H**). To examine the effects of PCCUR on collagen deposition and scar formation. Masson's trichrome staining was conducted 14 days postinjury (**Figure 7I and S18A**). Wounds displayed sparse and disorganized collagen fibers in the control and PCCUR groups; however, the groups treated with PCCUR under laser irradiation presented considerably increased deposition of collagens. Notably, the PCCUR + L-1 + L-2 treatment group exhibited neater collagen alignment in wounds compared to the PCCUR + L-1 group, which received only the first irradiation (0.5 W/cm²) and not the subsequent 0.3 W/cm² irradiation. Furthermore, newly formed hair follicles and sebaceous gland-like structures were visible in the PCCUR + L-1 + L-2 group. Overactivated myofibroblasts not only increase mechanical stress at the wound site but also secrete excessive amounts of collagen, ultimately leading to the development of pathological scars 110. α-SMA immunofluorescence was performed to identify activated fibroblasts in the wound (**Figure 7J and S18B**). A significant reduction in fibroblast activation was observed in wounds treated with two laser irradiations compared with those receiving only the first irradiation. These results suggest that following initial bacterial elimination through high-energy laser irradiation, PCCUR supports tissue regeneration and remodeling. Moreover, the second low-intensity laser irradiation during the proliferative phase appears to promote scarless wound healing by regulating fibroblast activation, which is driven primarily by the photodynamic capabilities of PCCUR.

To investigate the mechanism by which PCCUR modulates inflammation *in vivo*, RNA sequencing (RNA-seq) analysis was performed on wound tissues harvested 7 days postinjury from the control group and the PCCUR + L-1 group. The heatmap displayed in **Figure S19A** illustrates the clustering of DEGs between the two groups. By applying thresholds of |log_2_fold-change| ≥ 1 and a Q value ≤ 0.05, we identified 828 DEGs in the PCCUR + L-1 group compared with the control group, consisting of 515 upregulated and 313 downregulated genes (**Figure S19B**). GO analysis revealed that the DEGs were predominantly associated with terms such as neutrophil extravasation, leukocyte migration involved in the inflammatory response, C-X-C chemokine receptor binding, and neutrophil activation involved in the immune response (**Figure 7K**). Additionally, KEGG pathway analysis revealed that these DEGs were enriched in inflammation-related pathways, including the IL-17 signaling pathway, nuclear factor kappa B (NF-κB) signaling pathway, TNF signaling pathway, and Toll-like receptor signaling pathway (**Figure 7L**). Compared with that in the control group, the expression of 20 genes involved in the IL-17 signaling pathway was significantly decreased following PCCUR + L-1 treatment, suggesting a marked attenuation of the inflammatory response, which promotes wound healing (**Figure 7M**). The genes whose expression was most notably downregulated were IL-17A and IL-17F. Within the IL-17 family, IL-17A and IL-17F have been widely shown to exhibit proinflammatory properties 111. Specifically, IL-17A is known to drive neutrophil-mediated inflammation, which can hinder the wound healing process 112. Recent clinical trials have confirmed that anti-IL-17 monoclonal antibodies are effective in treating inflammatory skin diseases 113, 114. These findings are consistent with our results, where treatment with PCCUR + L-1 effectively reduced the inflammatory response and promoted wound healing by inhibiting the expression of IL-17A and IL-17F. Similarly, we observed a downregulation of 21 genes associated with the NF-κB signaling pathway (**Figure S20**). The NF-κB signaling pathway serves as a key regulatory switch for initiating proinflammatory and pro-oxidative reactions, regulating the expression of various inflammatory cytokines and chemokines. In this study, the photodynamic and photothermal actions of PCCUR, along with the anti-inflammatory characteristics of CE, collaboratively regulate inflammatory signaling pathways such as the IL-17 signaling pathway, alleviating infections and controlling inflammatory responses, thereby accelerating the wound healing process.

Postoperative melanoma patients are particularly prone to complications, including wound infections, impaired healing, and hypertrophic scarring 115-117. These complications are often associated with full-thickness skin defects and chronic inflammation caused by extensive tumor excision 11, 118. Consequently, persistent chronic inflammation leads to excessive proliferation of oncogenic cells, thereby inducing the recurrence of tumors 13. Our findings highlight the potential of PCCUR as an innovative treatment agent for addressing wound infections and inflammation, facilitating wound healing, and inhibiting scarring. In contrast to prior studies that focused only on wound infections or scar hypertrophy, our approach involves adjusting the laser power to meet specific therapeutic needs at different stages of wound healing 119, 120. During the inflammatory phase, high-intensity lasers have synergistic PDT and PTT effects to combat infections. In the remodeling phase, low-intensity lasers exhibit photodynamic characteristics that help prevent scar formation. Additionally, PCCUR possesses intrinsic anti-inflammatory properties and promotes cell proliferation and migration, which further aids in wound healing. These attributes may offer a promising avenue for the prevention of tumor recurrence. These findings present compelling evidence for the use of PCCUR hydrogels in the management of infected wounds.

### Effects of PCCUR on hypertrophic scars in rabbits

**Figure 8:**
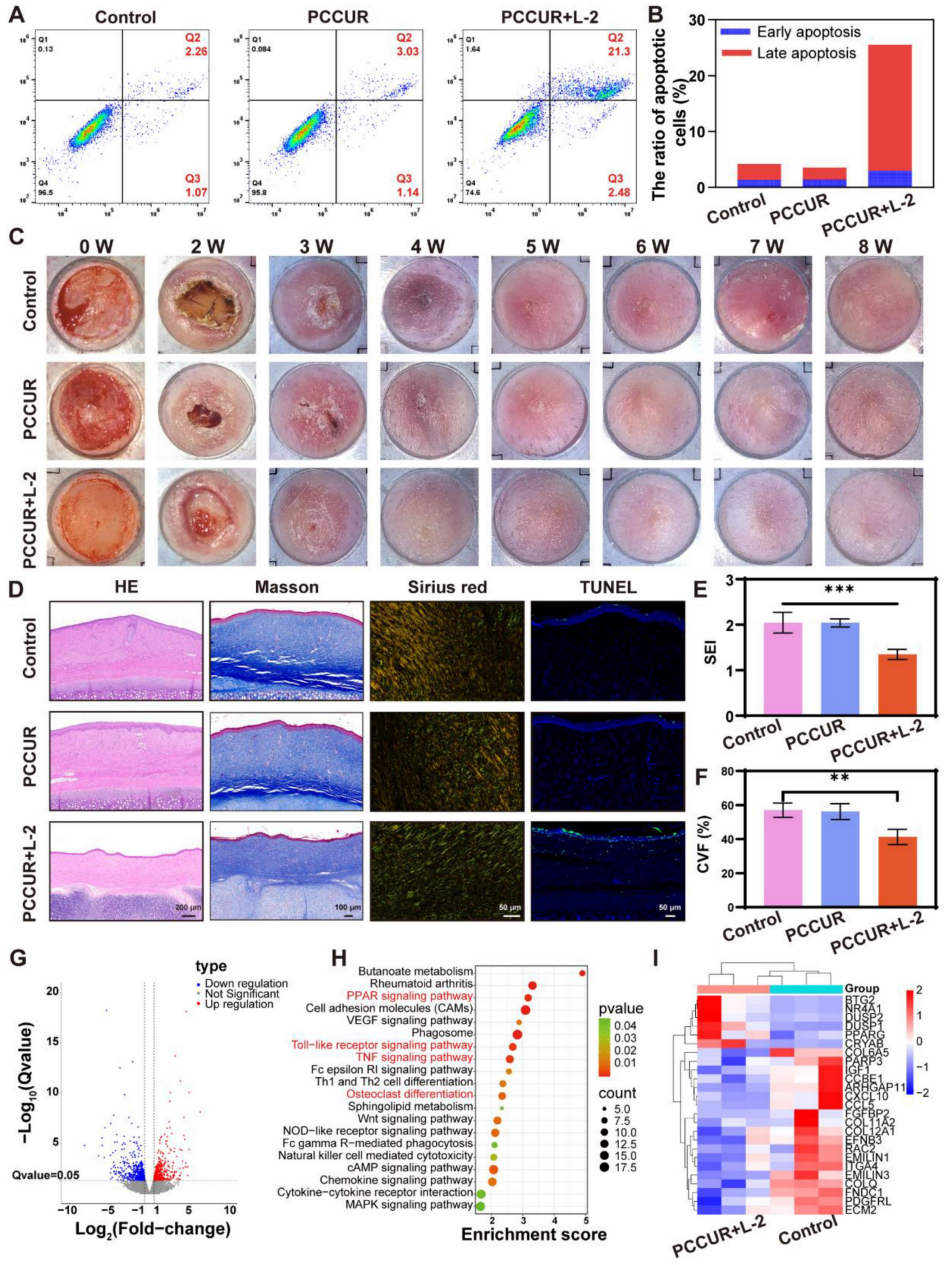
** Efficacy of PCCUR on hypertrophic scar models in rabbit ears.** (A) Flow cytometry results indicating the apoptosis of HSFs, along with (B) the corresponding quantitative analysis of apoptotic cells. (C) Visual representation of changes in the appearance of the ears of rabbits following different treatments. (D) Histological evaluations showing H&E staining, Masson's trichrome staining, Sirius red staining (observed under polarized light), and TUNEL immunofluorescence staining for each treatment group. The quantitative assessments included the (E) SEI derived from H&E staining and the (F) CVF measured from Masson's trichrome staining. (G) Volcano plots showing DEGs identified in the PCCUR + L-2 group versus the control group. (H) Enriched KEGG pathways associated with the identified DEGs. (I) Heatmap displaying scar formation-related genes based on RNA-seq data. L-2 (660 nm, 0.3 W/cm^2^). The data are presented as the means ± SDs (n = 3 or 4). **P < 0.01 and ***P < 0.001.

To elucidate the antiscar mechanism of PCCUR, we conducted an *in vitro* study *via* flow cytometry to assess its capacity to induce apoptosis in HSFs. As depicted in **Figure 8A and B**, both the control and the PCCUR groups maintained over 90% cell viability, confirming the superior biocompatibility of the hydrogel. Upon exposure to low-intensity laser (L-2, 660 nm, 0.3 W/cm^2^) irradiation, the PCCUR + L-2 group presented early and late apoptosis rates of 2.98 ± 0.44% and 22.53 ± 1.50%, respectively. On the basis of the established evidence that PCCUR is capable of generating singlet oxygen under conditions of low-intensity light exposure without photothermal effects, the apoptosis of HSFs is clearly mediated by PDT. Considering the variability of scar formation in murine models, we constructed a hypertrophic scar model in rabbit ears to better replicate human scar characteristics (**Figure 8C**). Macroscopic examination revealed complete re-epithelialization of all the wounds within 3 weeks postoperatively. After 5 weeks of treatment, the control group and the PCCUR group still presented rigid, mildly erythematous, and elevated scars. In contrast, the scars in the PCCUR + L-2 treatment group appeared paler and flatter. H&E staining further confirmed these observations, revealing a significant reduction in the scar elevation index (SEI) from 2.02 to 1.30 in the PCCUR + L-2 group, indicating the efficacy of the treatment (**Figure 8D and E**).

To evaluate collagen deposition, we performed Masson's trichrome staining on hypertrophic scar tissue (**Figure 8D and F**). The control group displayed coarse and irregularly distributed collagen fibers. There was no substantial improvement in collagen deposition following treatment with PCCUR in the absence of laser therapy. However, the PCCUR + L-2 group exhibited an orderly arrangement of collagen fibers within the scar tissue after treatment. Analysis of the collagen volume fraction (CVF) derived from Masson's trichrome staining revealed that the PCCUR + L-2 group had the lowest CVF of 41.30 ± 4.44%, whereas the control group had a CVF value of 57.03 ± 4.20%, indicating marked suppression of collagen deposition. Sirius Red staining provided further insights into the distribution of type I collagen and type III collagen. The control group predominantly displayed yellow-colored collagen fibers, which is indicative of a major accumulation of type I collagen. In the PCCUR + L-2 group, there was a significant reduction in the ratio of type I to type III collagens, indicating a significant improvement in collagen deposition. Moreover, TUNEL analysis was employed to evaluate the *in vivo* apoptosis of HSFs. Compared with the other groups, the PCCUR + L-2 group presented a significant increase in the percentage of TUNEL-positive cells.

To further explore the molecular mechanisms underlying the antiscar effects of PCCUR combined with laser irradiation, a comprehensive transcriptomic analysis was performed. The volcano plot, with criteria of a |log_2_fold-change| ≥ 0.585 and a Q value ≤ 0.05, revealed 1143 DEGs between the PCCUR + L-2 and control groups, including 519 upregulated and 624 downregulated genes (**Figure 8G**). GO enrichment analysis revealed that these DEGs were involved primarily in biological processes such as lipid digestion, extracellular matrix (ECM) binding, collagen binding, and the regulation of cell activation, highlighting the significant role of PCCUR + L-2 therapy in improving ECM deposition and remodeling during scar formation (**Figure S21**). Additionally, KEGG enrichment analysis revealed notable enrichment of pathways, including the peroxisome proliferator-activated receptor (PPAR) signaling pathway, the Toll-like receptor signaling pathway, the TNF signaling pathway, and the osteoclast differentiation signaling pathway, in the PCCUR + L-2 group compared with the control group (**Figure 8H**). Research by Hoerst et al. indicated that activation of the PPARγ signaling pathway induces reprogramming of myofibroblasts, as demonstrated by a marked decrease in the expression of α-SMA and ECM components, thereby initiating tissue remodeling 121. Previous studies have shown that prolonged overactivation of myofibroblasts, abnormal collagen synthesis and deposition, and increased mechanical stress are among the complex factors that ultimately lead to scar formation 12, 122. Furthermore, genes intimately linked to scar formation according to the RNA-seq data are clustered in **Figure 8I**. Relative to the control group, the PCCUR + L-2 treatment group presented upregulated expression of genes, including DUSP1, DUSP2, PPARG, and CRYAB, whereas the expression of genes, such as COL6A5, FNDC1, ARHGAP11A, and ECM2, was significantly downregulated. These findings are consistent with recent studies reporting an inverse relationship between ECM pathway-related gene expression and PPARG levels in human scleroderma skin tissue 123. These findings suggest that PCCUR may inhibit scar formation by activating the PPAR signaling pathway through the upregulation of PPARG expression induced by phototherapy.

In summary, our study demonstrated that PCCUR combined with low-intensity laser irradiation significantly diminishes the formation of hypertrophic scars in a rabbit ear model. This therapy promotes the apoptosis of HSF cells and markedly enhances collagen deposition and remodeling, thereby effectively inhibiting scar formation. These findings emphasize that PCCUR + L-2 therapy may regulate the expression of genes related to scar formation by activating the PPAR signaling pathway, providing new insights into the molecular basis of scar prevention. Future research should focus on clarifying the principal molecular targets involved in PCCUR + L-2 therapy to increase its clinical efficacy.

In this pioneering investigation, we present the application of an innovative PCCUR hydrogel for the holistic management of melanoma. Our clinical findings underscore a significant risk of postsurgical complications in melanoma patients, necessitating cutting-edge therapeutic intervention. PCCUR, which integrates PDA-NPs and CE, effectively targets malignant cells through phototherapy while promoting wound healing. Its distinct characteristics, such as injectability and biodegradability, ensure the sustained release of therapeutic agents, thereby enhancing the efficacy of phototherapy. In the inflammatory phase, PCCUR-based PDT and PTT synergize to combat wound infections and trigger ICD, releasing tumor antigens that serve as immune stimulants and overcoming tumor heterogeneity. These antigens are captured by an adhesive hydrogel, which functions as an “antigen depot” to recruit DCs and activate antitumor immunity. The proliferation phase is marked by the hydrogel's promotion of anti-inflammatory effects, robust cell proliferation, and angiogenesis, which are crucial processes for achieving optimal wound healing. The remodeling phase is carefully managed with low-intensity laser phototherapy to modulate myofibroblast activity and collagen remodeling, preventing hypertrophic scarring. This integrated approach, guided by the 3R philosophy, addresses the critical aspects of postoperative management in a coordinated manner. The biocompatibility, scalability, and facile synthesis process of PCCUR underscore its significant translational potential as a breakthrough therapy for melanoma. This hydrogel system aligns with the skin protection principles advocated by the CACA technical guidelines for comprehensive cancer management, offering a transformative approach to skin protection in oncology. The integration of the 3R paradigm into the PCCUR design not only optimizes melanoma treatment technically but also marks a shift toward patient-centric, holistic cancer management. The extensive applicability of this strategy signals its potential to revolutionize skin-sparing therapies for a diverse range of malignancies beyond melanoma.

## Supplementary Material

Supplementary methods and figures.

Supplementary table 1.
