# Supplementary material for: Phase-specific and laser-modulated polydopamine-chlorella-curdlan hydrogels: pioneering a melanoma integrative therapy
Source: Theranostics. 2025 Jun 23;15(15):7627–52. doi: 10.7150/thno.113417 (PMC12316037; doi:10.7150/thno.113417)
Supplement: Supplementary file 1 — Supplementary methods and figures. [file thnov15p7627s1.pdf]

Supplementary Material

Phase-Specific and Laser-Modulated Polydopamine-Chlorella-Curdlan Hydrogels: Pioneering a Melanoma Integrative Therapy

Zhenzhen Yan[1], Tinglin Zhang[2, 5], Yuxiang Wang[1], Chao Ji[1], Heru Wang[3], Yicheng Ma[1], Jingzhu Li[1], Yixin Wu[1], Zhi Liu[4], Jingnan Xun[1], Xiaowan Fang[1], Yongjun Zheng[1, *], Shizhao Ji[1, *], Shichu Xiao[1, *], and Jie Gao[2, 5, *]

1. Department of Burn Surgery, The First Affiliated Hospital of Naval Medical University, Shanghai 200433, P. R. China

2. Changhai Clinical Research Unit, Shanghai Changhai Hospital, Naval Medical University, Shanghai 200438, P. R. China

3. Neurovascular Center, The First Affiliated Hospital of Naval Medical University, Shanghai 200433, P. R. China

4. Department of Dermatology, The First Affiliated Hospital of Naval Medical University, Shanghai 200433, P. R. China

5. Shanghai Key Laboratory of Nautical Medicine and Translation of Drugs and Medical Devices, Shanghai 200433, P. R. China

Zhenzhen Yan, Tinglin Zhang, Yuxiang Wang and Chao Ji contributed equally to this paper.

*Corresponding authors:

Phone: 86-021-3116 1825.

E-mail addresses: gaojiehighclea@smmu.edu.cn (J. Gao); huangzhuoxiao4@hotmail.com (S. Xiao); shizhaoji2022@163.com (S. Ji); smmuzhengyongjun@163.com

(Y. Zheng).

Methods.

Synthesis of polydopamine nanoparticles (PDA-NPs)

PDA-NPs were synthesized following a previously established protocol. Briefly, 1 mL of ammonia (25%) solution was mixed with 90 mL of deionized water and 40 mL of ethanol (70/30% v/v) and prestirred for 30 min at 25 °C. Dopamine hydrochloride (950 mg, 10 mmol), which was predissolved in 20 mL of deionized water, was added to this mixture under light stirring. The reaction was maintained at 25 °C with gentle stirring for 24 h. The resulting product was collected by centrifugation at 15,000 rpm for 15 min, washed three times with deionized water, and freeze-dried for subsequent use. The characteristics of the PDA-NPs were analyzed *via* dynamic light scattering (DLS) and transmission electron microscopy (TEM, JEOL JEM-F200, Tokyo, Japan).

Swelling rate analysis

The swelling behavior of the CUR, CCUR, and PCCUR hydrogels was examined in phosphate-buffered saline (PBS). Briefly, 200 μL of each hydrogel was initially weighed (W_0) and immersed in sufficient PBS at 37 °C. At predetermined intervals, the hydrogels were removed and reweighed (W_t). The swelling ratio was calculated *via* Equation (1):

$$\text{Swelling ratio (\%)} = \left(\frac{W_t - W_0}{W_0}\right) \times 100\% \qquad \text{Equation (1)}$$

Degradation behavior analysis

To evaluate the degradation profile of the CUR, CCUR, and PCCUR hydrogels, 200 μL samples of each hydrogel were placed into 24-well plates and immersed in sufficient PBS with constant agitation. The hydrogels were lyophilized at specific time intervals, and their weights (W_t) were recorded. The initial weight on day 0 (W_0) was used as the baseline. The degradation rate was calculated according to Equation (2):

$$\text{Degradation rate (\%)} = \left(\frac{W_0 - W_t}{W_0}\right) \times 100\% \qquad \text{Equation (2)}$$

Protein Adsorption of Hydrogels

To assess the protein adsorption capacity of the hydrogels, 200 μL of each prepared

hydrogel was incubated with 100 μL of fetal bovine serum (FBS) in a shaking incubator (37 °C, 100 rpm) for 24 h. After incubation, the samples were centrifuged at 10,000 rpm for 10 min to separate the supernatant. The FBS concentration in the supernatant (C_t) was measured *via* a Bradford protein assay kit (P0006, Biyuntian, Shanghai, China), with the control group (C_0) lacking hydrogel used for comparison. The percentage of the adsorbed protein content was quantified *via* Equation (3):

$$\text{Adsorbed protein content (\%)} = \left(\frac{C_0 - C_t}{C_0}\right) \times 100\% \qquad \text{Equation (3)}$$

Hemolysis assay

Fresh blood samples were obtained from healthy C57BL/6 mice and mixed with ethylenediaminetetraacetic acid to prevent coagulation. Subsequently, the erythrocytes were washed with PBS to remove hemoglobin. The washed red blood cells were then resuspended in PBS to achieve a final concentration of 10%. An equal volume of hydrogel extract was introduced to the erythrocyte suspension. Ultrapure water served as a positive control, whereas PBS served as a negative control. The mixtures were incubated at 37 °C in a constant-temperature incubator for 1 h. After incubation, the samples were centrifuged at 1000 rpm for 5 min. Following centrifugation, photographs were taken to visually assess the degree of hemolysis. The optical density (OD) of the supernatant at 545 nm was subsequently measured via a plate reader. The hemolysis rate (HR) was calculated via the following formula:

$$\text{Hemolysis rate (\%)} = \left(\frac{OD_S - OD_{nc}}{OD_{pc} - OD_{nc}}\right) \times 100\% \qquad \text{Equation (4)}$$

where OD_s, OD_{nc}, and OD_{pc} represent the OD values in the hydrogels, PBS, and ultrapure water, respectively.

***In vitro* induction of immunogenic cell death (ICD)**

The induction of ICD in B16F10 tumor cells was evaluated as described below. To evaluate this, several parameters, including the surface expression of calreticulin (CRT), the extracellular release of high-mobility group box 1 (HMGB1), and adenosine triphosphate (ATP) secretion, were measured. B16F10 cells were seeded at a density of 5×10^4 cells per well in 12-well plates and cultured for 12 h. Subsequently, different hydrogels or PBS (as a control) were administered for 24 hours. In the laser irradiation

groups, the cells were exposed to a 660 nm laser at 0.5 W/cm^2 for 5 min, followed by an additional 24 h of incubation. For CRT expression assessment, the cells were fixed with 4% paraformaldehyde (PFA) for 30 min and permeabilized with 0.2% Triton X-100 for 10 min. An anti-CRT antibody (Proteintech, Wuhan, China) was added, and the samples were incubated overnight at 4 °C. This was followed by incubation with an Alexa Fluor® 488-conjugated secondary antibody (Thermo Fisher Scientific, Massachusetts, USA) for 1 h at room temperature. The cells were then stained with DAPI (Biyuntian, China) for 20 min and examined *via* laser scanning confocal microscopy (CLSM) (Olympus, FV1000, Tokyo, Japan). To quantify the release of HMGB1 and ATP, culture supernatants were collected 24 h after laser irradiation. The concentrations of HMGB1 and ATP were determined *via* ELISA kits (Elabscience, Wuhan, China) and ATP assay kits (Beyotime, Shanghai, China), respectively.

Supplementary Figures

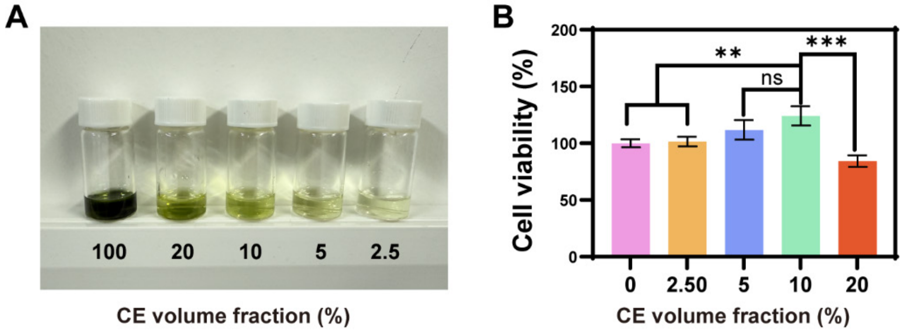

Figure S1. (A) Visualization of CE dilutions with different volume fractions. (B) Cytotoxicity of different volume fractions of CE. The data are presented as the means ± SDs (n = 3).

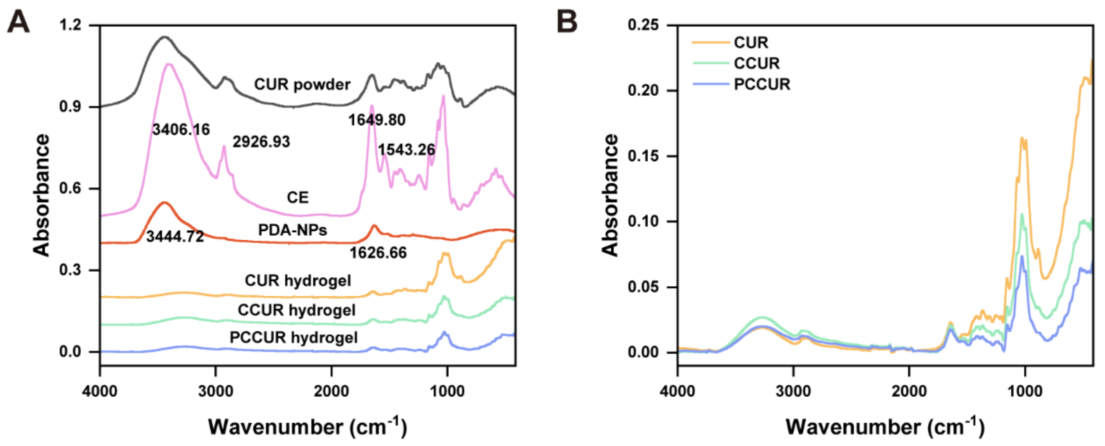

Figure S2. (A) Fourier transform infrared spectroscopy (FTIR) of CUR powder, CE, PDA-NPs, and three types of hydrogels. (B) FTIR spectra of CUR, CCUR, and PCCUR hydrogels.

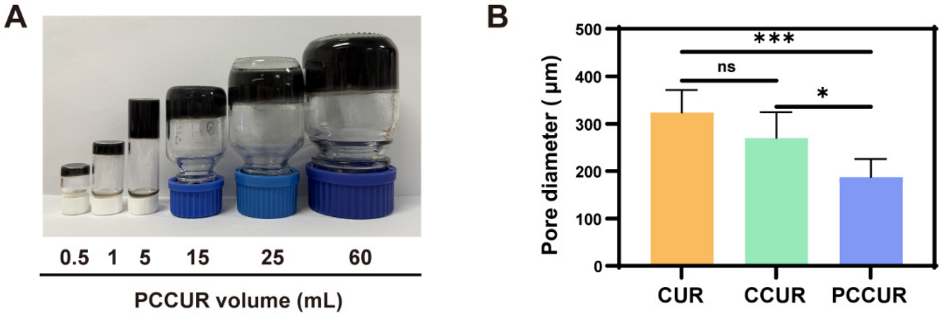

Figure S3. (A) High-volume preparation of PCCUR hydrogels. (B) Pore size of the hydrogels. The data are presented as the means ± SDs (n = 3). ns (not significant), $^{*}P < 0.05$, $^{***}P < 0.001$.

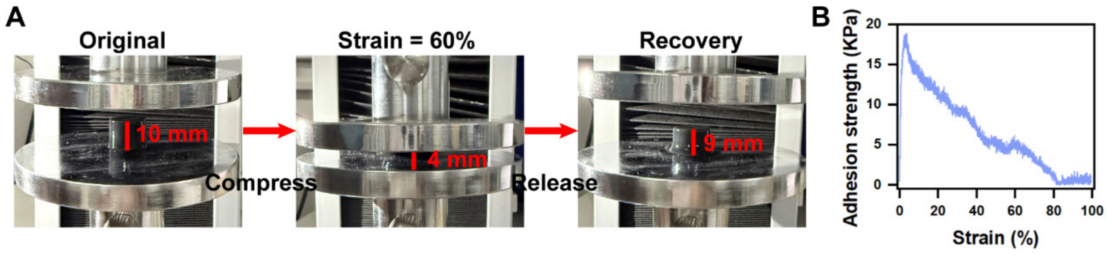

Figure S4. (A) Visual illustration of PCCUR under 60% maximum compression strain. (B) Adhesion strength of the PCCUR hydrogel.

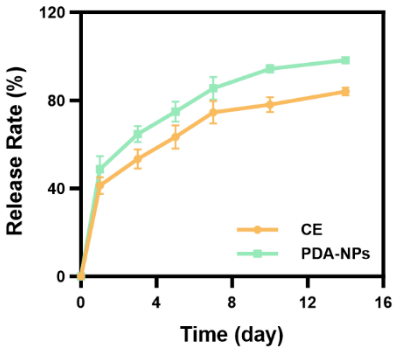

Figure S5. Release curves of CE and PDA-NPs from PCCUR hydrogel. The data are presented as the means ± SDs (n = 3).

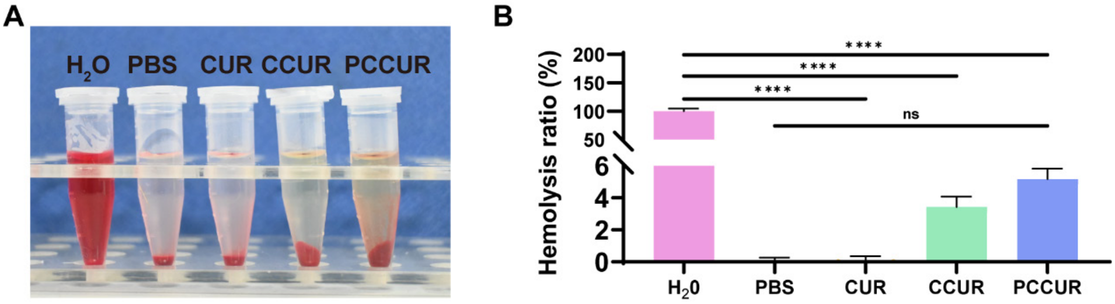

Figure S6. (A) Representative pictures of *in vitro* hemolysis experiments. (B) Hemolysis rate of the hydrogels. The data are presented as the means ± SDs (n = 3). ns (not significant), ****$P < 0.0001$.

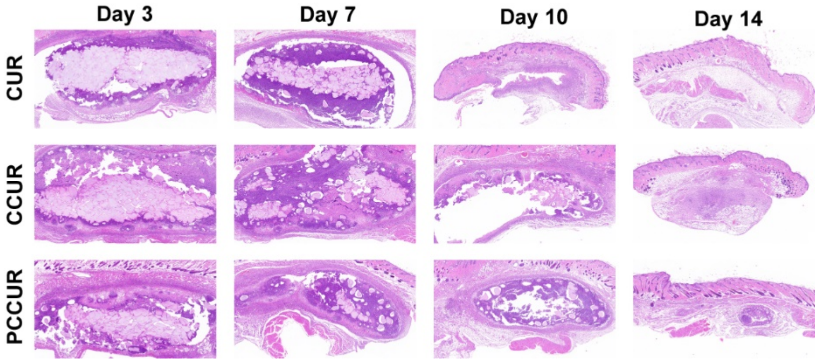

Figure S7. Representative HE stained images of CUR hydrogel, CCUR hydrogel, and PCCUR hydrogel and surrounding tissues after being implanted in subcutaneous for 3 days, 7 days, 10 days, and 14 days.

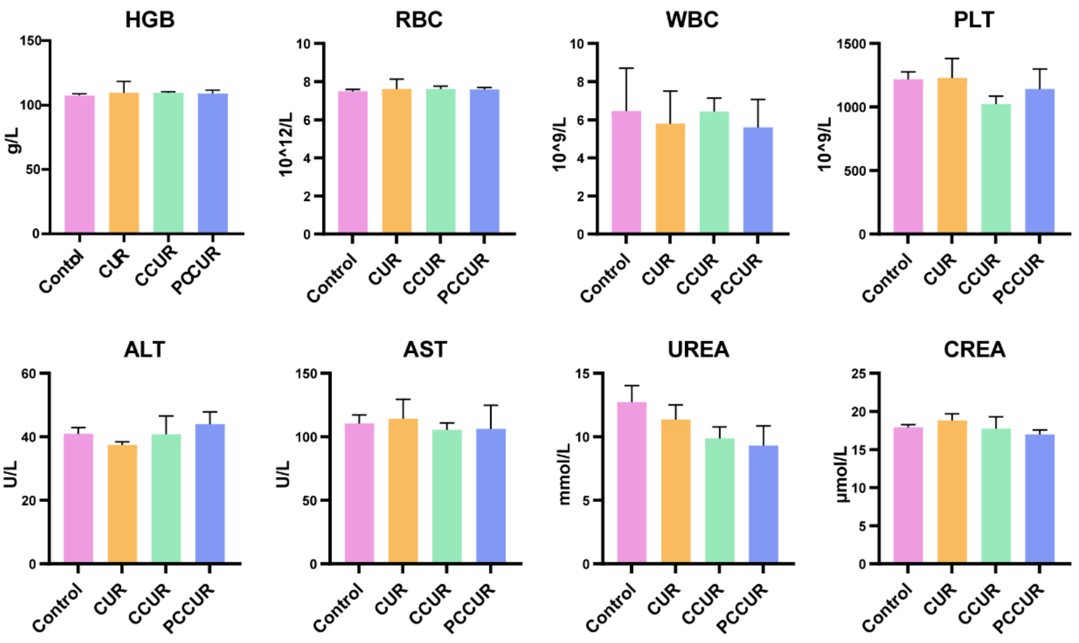

Figure S8. Results of routine blood and liver and kidney function tests in mice implanted subcutaneously with different hydrogels. The data are presented as the means ± SDs (n = 3). HGB (Hemoglobin), RBC (Red blood cell), WBC (White blood cell), PLT (Platelet), ALT (Alanine aminotransferase), AST (Aspartate aminotransferase), UREA (Urea), CREA (Creatinine).

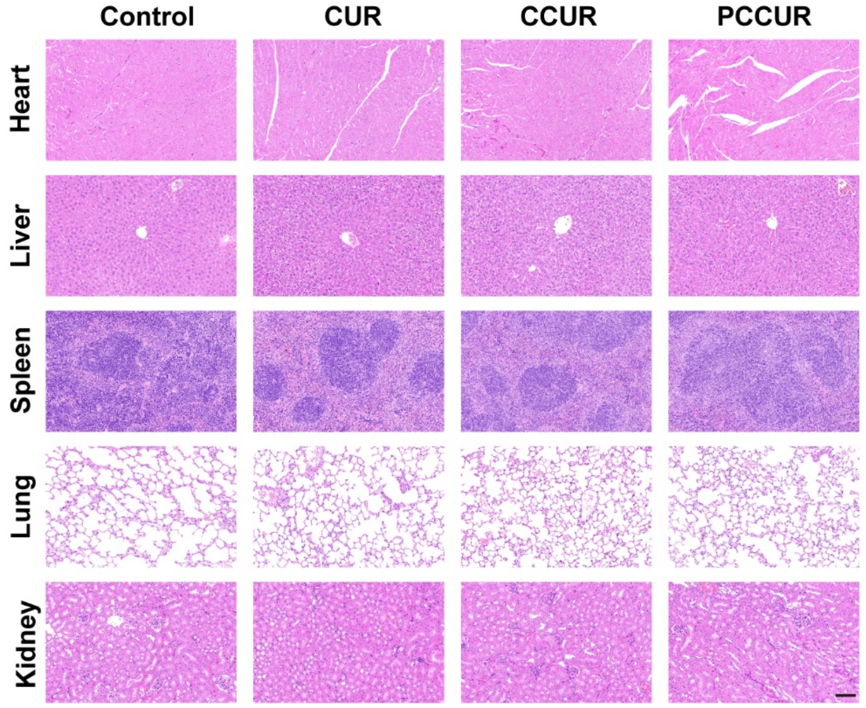

Figure S9. H&E staining of tissue sections from the main organs (heart, liver, spleen, lungs and kidneys) after being implanted subcutaneously with different hydrogels.

Scale bars = 100 μm.

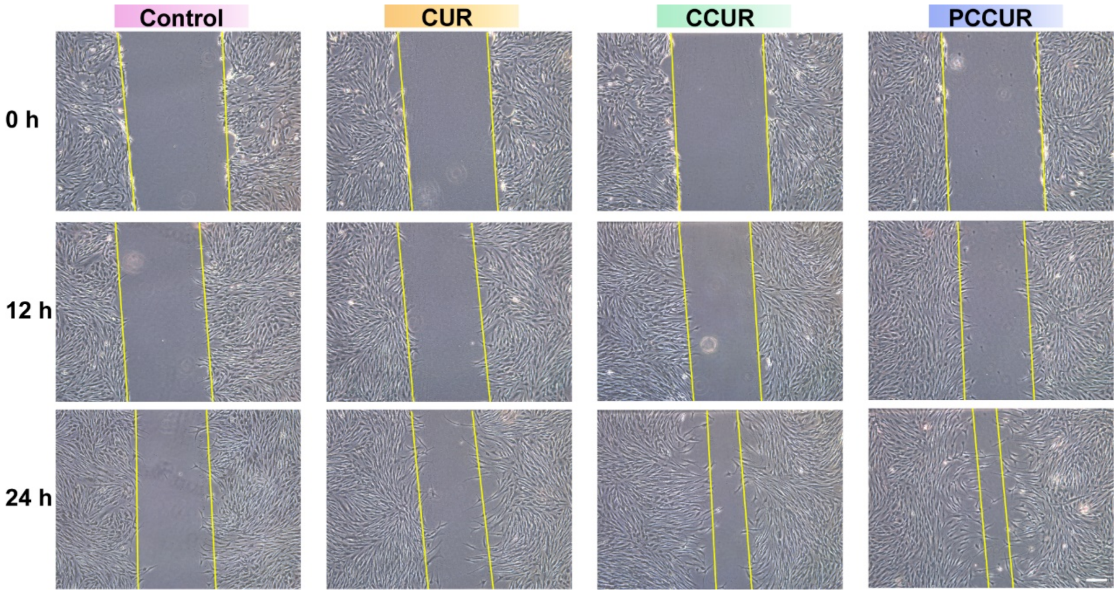

Figure S10. Representative images of the migration area of HFBs at different time points posttreatment (scale bars: 200 μm).

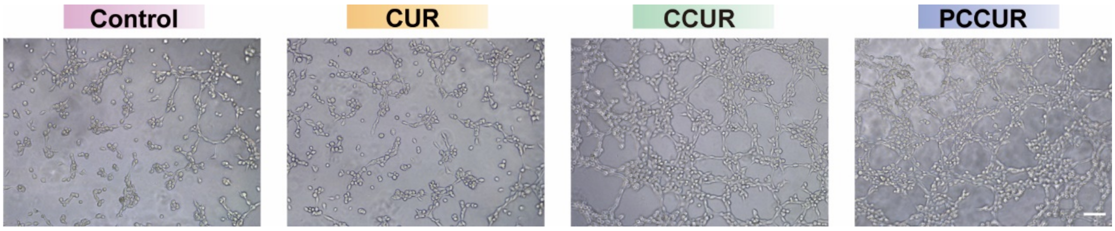

Figure S11. Representative images from the tube formation assay of HUVECs (scale bars: 100 μm).

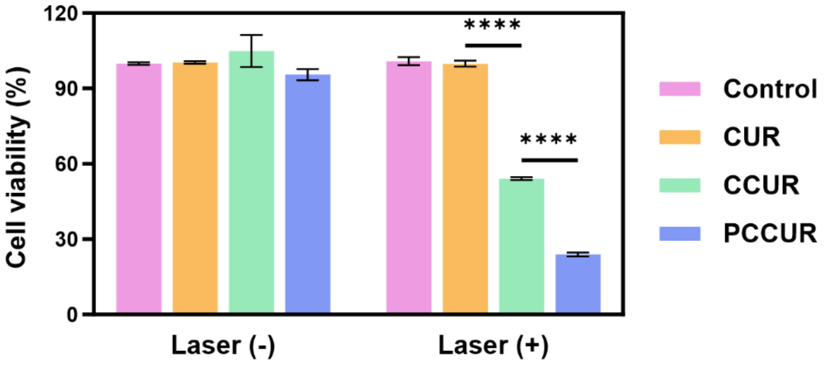

Figure S12. Viability of B16F10 cells after different treatments. The data are presented as the means ± SDs (n = 3). ****P < 0.0001. Lase (660 nm, 0.5 W/cm^2).

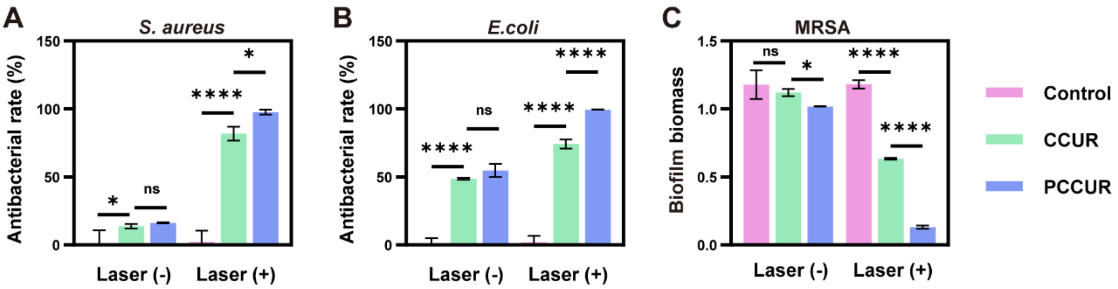

Figure S13. Quantitative analysis of plate colony formation of (A) *S. aureus* and *E. coli* (B). (C) Quantitative evaluation of MRSA biofilms via crystal violet staining. The data are presented as the means ± SDs (n = 3). ns (not significant), *P < 0.05, ****P < 0.0001. Lase (660 nm, 0.5 W/cm^2).

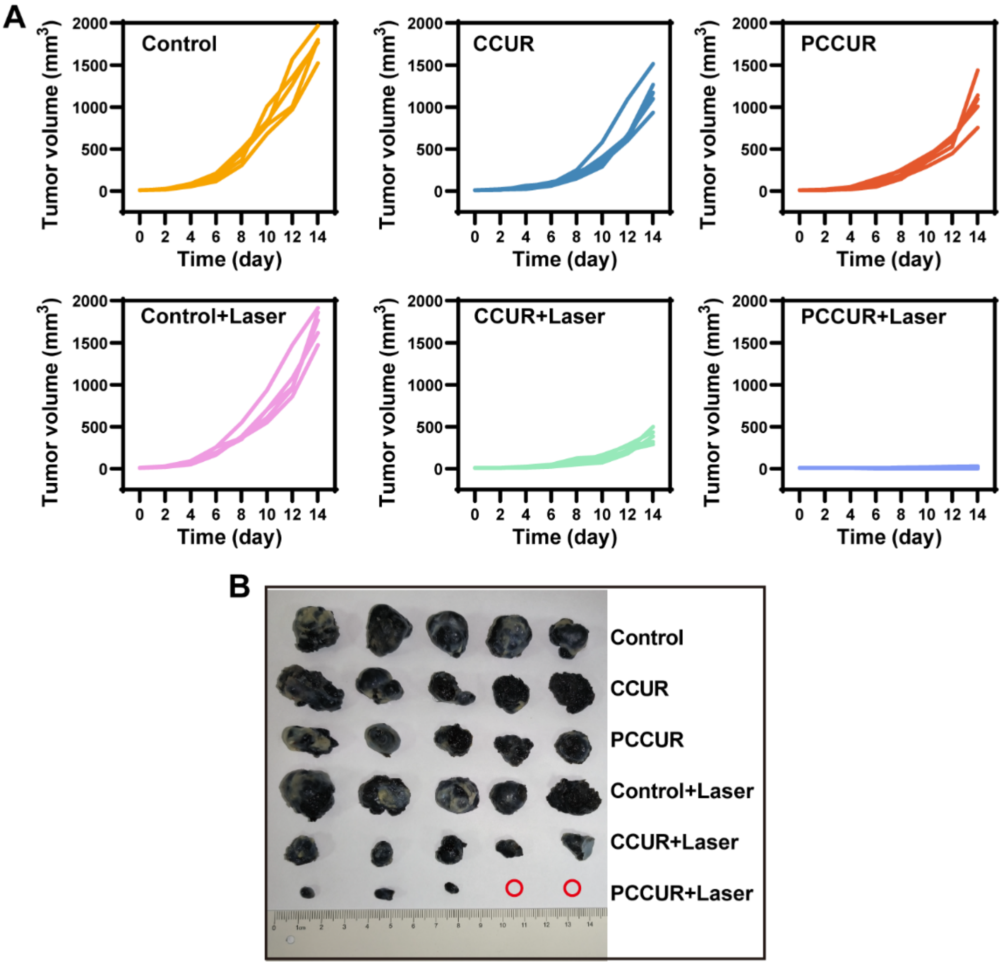

Figure S14. (A) Tumor growth curves for each mouse across the various treatment groups (n = 5). (B) Representative images of excised tumors after different treatments. Red circles represent no tumor recurrence. Lase (660 nm, 0.5 W/cm^2).

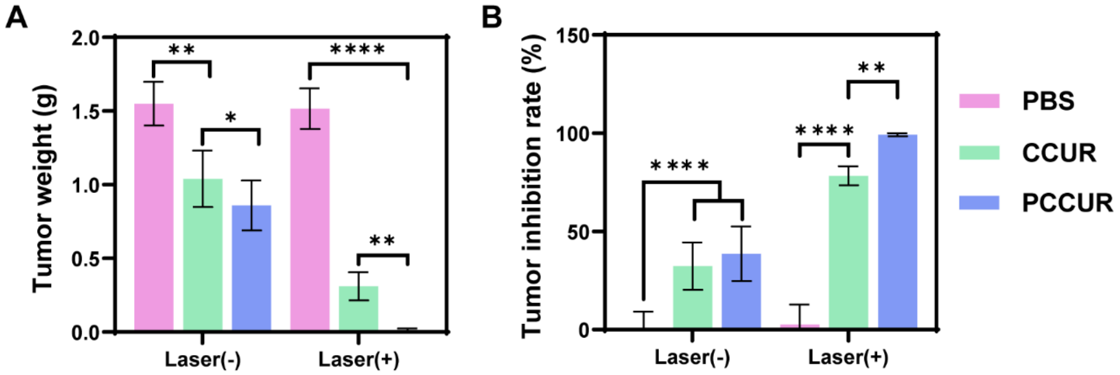

Figure S15. (A) Tumor weights of the mice at the study endpoint. (B) Inhibition rate of tumor volume at the study endpoint. The data are presented as the means ± SDs (n = 5). *$P < 0.05$, **$P < 0.01$, ****$P < 0.0001$. Lase (660 nm, 0.5 W/cm^2).

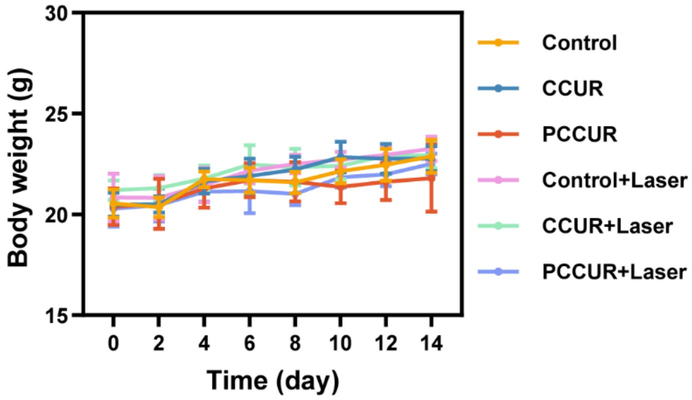

Figure S16. Body weight variations across different treatment regimens. The data are presented as the means ± SDs (n = 5). Lase (660 nm, 0.5 W/cm^2).

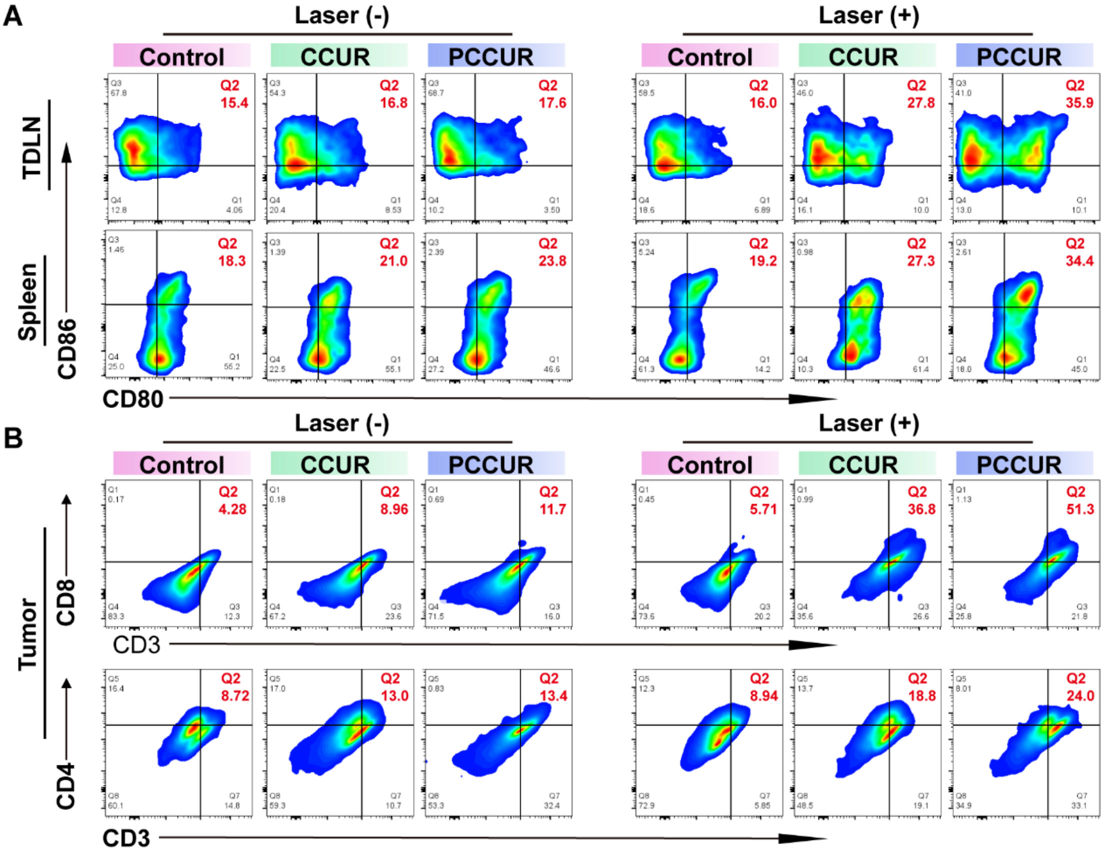

Figure S17. *In vivo* activation of antitumor immunity. (A) Flow cytometry analysis showing mature DCs in the TDLN and the spleen. (B) Flow cytometry plot illustrating the presence of $CD3^{+}$ $CD8^{+}$ T cells and $CD3^{+}$ $CD4^{+}$ T cells in tumors. Lase (660 nm, 0.5 W/cm^2).

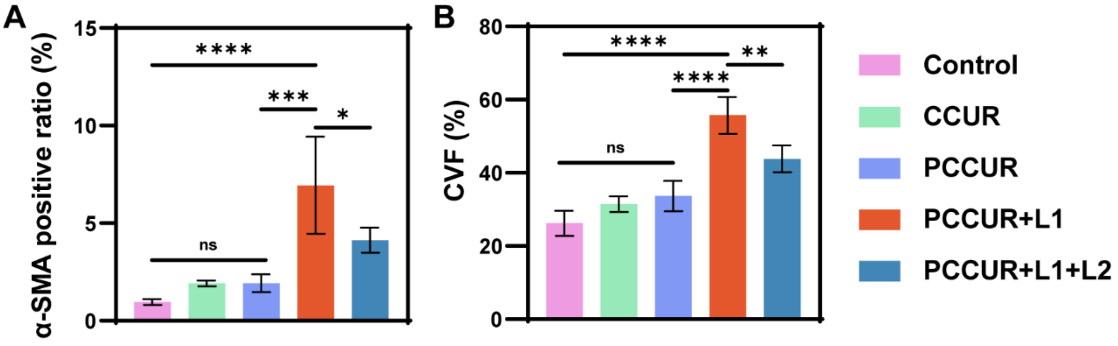

Figure S18. Quantification of (A) collagen deposition and (B) α-SMA expression in wound tissue on day 14. L-1 (660 nm, 0.5 W/cm^2), and L-2 (660 nm, 0.3 W/cm^2). The data are presented as the means ± SDs (n = 4). ns (not significant), *$P < 0.05$, **$P < 0.01$, ***$P < 0.001$, ****$P < 0.0001$.

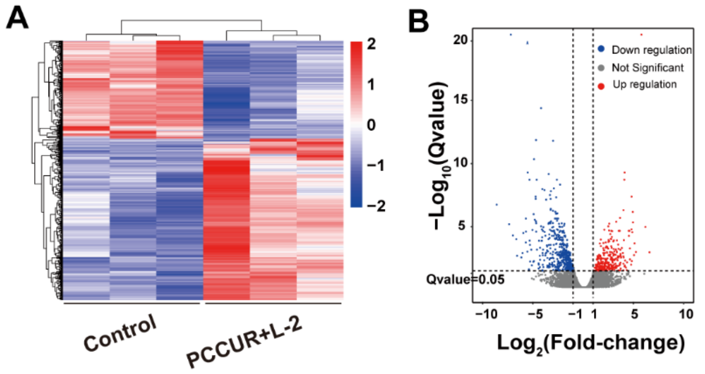

Figure S19. (A) Heatmap analysis of DEGs in the PCCUR + L-1 group compared with the control group on day 7 posttreatment. (B) Volcano plot depicting DEGs between the PCCUR + L-1 and control groups, highlighting significant changes in expression.

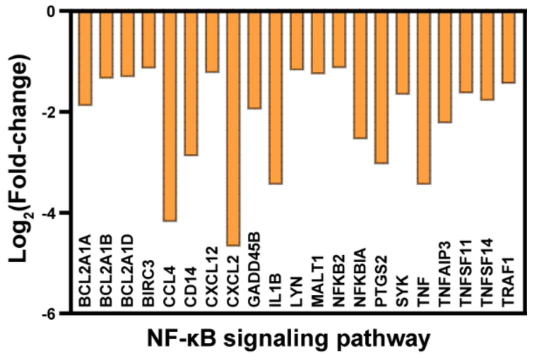

Figure S20. Changes in the expression levels of DEGs associated with the NF-κB signaling pathway following treatment with PCCUR + L-1. DEGs (differentially expressed genes), NF-κB (nuclear factor kappa B). L-1 (660 nm, 0.5 W/cm^2).

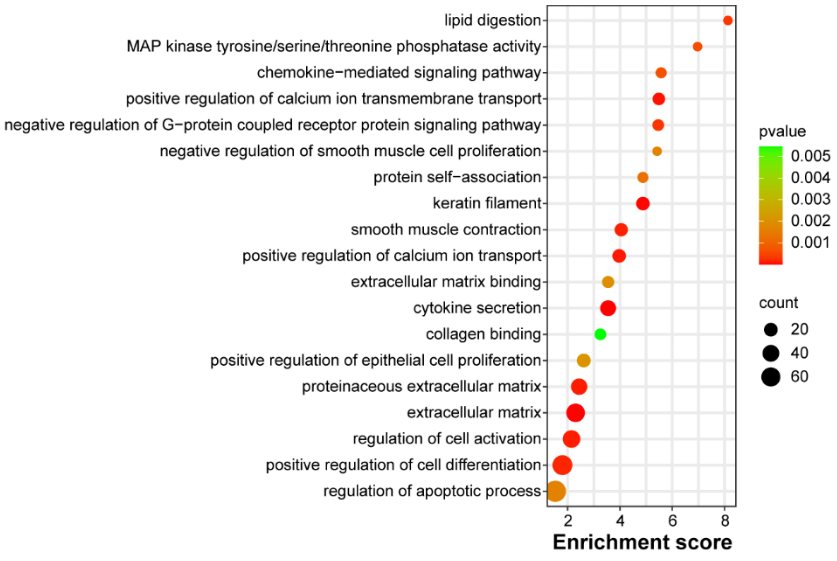

Figure S21. Gene Ontology (GO) analysis of differentially expressed genes (DEGs) between the PCCUR + L-2 and control groups. L-2 (660 nm, 0.3 W/cm^2).
